# Potent Stimulation of the Androgen Receptor Instigates a Viral Mimicry Response in Prostate Cancer

**DOI:** 10.1158/2767-9764.CRC-21-0139

**Published:** 2022-07-25

**Authors:** Mohammadreza Alizadeh-Ghodsi, Katie L. Owen, Scott L. Townley, Damien Zanker, Samuel P.G. Rollin, Adrienne R. Hanson, Raj Shrestha, John Toubia, Tessa Gargett, Igor Chernukhin, Jennii Luu, Karla J. Cowley, Ashlee Clark, Jason S. Carroll, Kaylene J. Simpson, Jean M. Winter, Mitchell G. Lawrence, Lisa M. Butler, Gail P. Risbridger, Benjamin Thierry, Renea A. Taylor, Theresa E. Hickey, Belinda S. Parker, Wayne D. Tilley, Luke A. Selth

**Affiliations:** 1 Dame Roma Mitchell Cancer Research Laboratories, Adelaide Medical School, The University of Adelaide, Adelaide, SA, Australia.; 2 Freemasons Centre for Male Health and Wellbeing, The University of Adelaide, Adelaide, SA, Australia.; 3 Cancer Evolution and Metastasis Program, Peter MacCallum Cancer Centre, Melbourne, Victoria, Australia.; 4 Sir Peter MacCallum Department of Oncology, The University of Melbourne, Parkville, Victoria, Australia.; 5 Flinders Health and Medical Research Institute, Flinders University, Bedford Park, SA, Australia.; 6 Freemasons Centre for Male Health and Wellbeing, Flinders University, Bedford Park, SA, Australia.; 7 Centre for Cancer Biology, University of South Australia and SA Pathology, Adelaide, SA, Australia.; 8 ACRF Cancer Genomics Facility, Centre for Cancer Biology, SA Pathology and University of South Australia, Frome Road, Adelaide, SA, Australia.; 9 Cancer Research UK Cambridge Institute, University of Cambridge, Cambridge, United Kingdom.; 10 Victorian Centre for Functional Genomics, Peter MacCallum Cancer Centre, Melbourne, Victoria, Australia.; 11 Monash Partners Comprehensive Cancer Consortium, Monash Biomedicine Discovery Institute Cancer Program, Prostate Cancer Research Group, Department of Anatomy and Developmental Biology, Monash University, Clayton, Victoria, Australia.; 12 Peter MacCallum Cancer Centre, University of Melbourne, Melbourne, Victoria, Australia.; 13 Cabrini Institute, Malvern, Victoria, Australia.; 14 Melbourne Urological Research Alliance (MURAL), Monash Biomedicine Discovery Institute Cancer Program, Department of Anatomy and Developmental Biology, Monash University, Clayton, Victoria, Australia.; 15 South Australian Health and Medical Research Institute, Adelaide, SA, Australia.; 16 Faculty of Health and Medical Sciences, The University of Adelaide, Adelaide, SA, Australia.; 17 ARC Centre of Excellence in Convergent Bio and Nano Science and Technology, University of South Australia, Frome Road, Adelaide, SA, Australia.; 18 Future Industries Institute, University of South Australia, Mawson Lakes, SA, Australia.; 19 Monash Partners Comprehensive Cancer Consortium, Monash Biomedicine Discovery Institute Cancer Program, Prostate Cancer Research Group, Department of Physiology, Monash University, Clayton, Victoria, Australia.

## Abstract

Inhibiting the androgen receptor (AR), a ligand-activated transcription factor, with androgen deprivation therapy is a standard-of-care treatment for metastatic prostate cancer. Paradoxically, activation of AR can also inhibit the growth of prostate cancer in some patients and experimental systems, but the mechanisms underlying this phenomenon are poorly understood. This study exploited a potent synthetic androgen, methyltestosterone (MeT), to investigate AR agonist-induced growth inhibition. MeT strongly inhibited growth of prostate cancer cells expressing AR, but not AR-negative models. Genes and pathways regulated by MeT were highly analogous to those regulated by DHT, although MeT induced a quantitatively greater androgenic response in prostate cancer cells. MeT potently downregulated DNA methyltransferases, leading to global DNA hypomethylation. These epigenomic changes were associated with dysregulation of transposable element expression, including upregulation of endogenous retrovirus (ERV) transcripts after sustained MeT treatment. Increased ERV expression led to accumulation of double-stranded RNA and a “viral mimicry” response characterized by activation of IFN signaling, upregulation of MHC class I molecules, and enhanced recognition of murine prostate cancer cells by CD8^+^ T cells. Positive associations between AR activity and ERVs/antiviral pathways were evident in patient transcriptomic data, supporting the clinical relevance of our findings. Collectively, our study reveals that the potent androgen MeT can increase the immunogenicity of prostate cancer cells via a viral mimicry response, a finding that has potential implications for the development of strategies to sensitize this cancer type to immunotherapies.

**Significance:**

Our study demonstrates that potent androgen stimulation of prostate cancer cells can elicit a viral mimicry response, resulting in enhanced IFN signaling. This finding may have implications for the development of strategies to sensitize prostate cancer to immunotherapies.

## Introduction

Prostate cancer cells are exquisitely dependent on androgens and the androgen receptor (AR) for growth and survival, which explains the efficacy of androgen deprivation therapy (ADT) as a treatment strategy for advanced prostate cancer. ADT involves various strategies to reduce circulating androgen levels and/or directly block AR activity. While almost all prostate tumors initially respond to ADT, the development of a therapy-resistant disease state, referred to as castration-resistant prostate cancer (CRPC), is inevitable. In the vast majority of cases, resistance to ADT is mediated by adaptive alterations to the AR signaling axis, highlighting addiction to this pathway as a hallmark of prostate cancer (1).

AR is a transcription factor that, upon binding to androgen, translocates from the cytoplasm to the nucleus and interacts with specific *cis*-regulatory elements (termed androgen response elements) on chromatin to regulate a gene expression program that promotes growth, survival, and metabolism of prostate cancer cells. The transcriptional output of AR can be influenced by a multitude of parameters, including hundreds of coregulators (2), epigenetic factors (3), and the concentration and composition of the androgenic milieu (4). Additional complexity arises from the evolution of AR signaling axis components during progression to CRPC. For example, direct changes to the *AR* gene (mutation, amplification, and rearrangements that result in AR splicing alterations) alter cellular responses to androgens, alternative ligands, and antiandrogens, collectively enabling high AR activity despite ongoing ADT (1).

Not surprisingly, most research on AR in prostate cancer has focused on its oncogenic functions. However, it is important to consider that in normal adult prostate epithelial cells the AR promotes cellular quiescence by preserving luminal differentiation and protein-secretory activity (1). This understanding may explain the decades-old observation that administration of high doses of testosterone can result in clinical responses in men with CRPC (5). This apparent paradox is supported by preclinical studies demonstrating that low androgen levels promote growth of prostate cancer whereas high androgen concentrations are growth inhibitory (6–8). The concept of therapeutic application of androgens in prostate cancer has culminated in recent clinical trials testing supraphysiologic levels of testosterone (SupraT), which have yielded promising results in a subset of patients (9–13). In the clinic, SupraT is combined with ADT such that patients are cycled between near-castrate and very high serum T levels, a treatment strategy referred to as bipolar androgen therapy (BAT; refs. 9–13).

A detailed understanding of the mechanism(s) by which androgens can elicit AR's growth-inhibitory activity is critical for the optimization of novel hormonal therapies. In this study, we investigated the mode of action of a synthetic androgen, 17α-methyl-testosterone (MeT), which is known to potently inhibit the growth of the LNCaP model of prostate cancer (14). MeT is an anabolic steroid developed in the 1930s that is used as a hormonal treatment in men and women (15). By dissecting MeT-induced transcriptional changes, we uncovered a novel response of prostate cancer cells to potent androgen stimulation. Specifically, we found that MeT downregulated DNA methyltransferases, resulting in DNA hypomethylation throughout the genome. This phenomenon was associated with increased expression of endogenous retrovirus transcripts, activation of IFN signaling, and enhanced immunogenicity of prostate cancer cells. Thus, our findings demonstrate that the potent androgen MeT can cause viral mimicry in prostate cancer cells, which may provide a basis for new targeted investigations into combining androgen therapies with immunotherapies.

## Materials and Methods

### Cell Lines and Cell Culture

The human prostate carcinoma cell lines LNCaP (RRID: CVCL_1379), VCaP (RRID: CVCL_2235), PC3 (RRID: CVCL_0035), 22Rv1 (RRID: CVCL_1045) and C4-2B (RRID: CVCL_4784) were obtained from the ATCC. LNCaP-V16D, LNCaP-MR49F, and CWR-R1-D567 have been described previously (16, 17). Luciferase-labelled RM1, a murine syngeneic model of bone-metastatic prostate cancer, has been described previously (18). C4-2B, 22Rv1, LNCaP, and LNCaP-V16D cells were maintained in RPMI1640 (Sigma-Aldrich) containing 10% FBS and 2 mmol/L l-Glutamine. PC3 cells were cultured in RPMI1640 containing 5% FBS and 2 mmol/L l-Glutamine. LNCaP-MR42D and LNCaP-MR49F were maintained in RPMI1640 containing 10% FBS, 10 μmol/L enzalutamide, and 2 mmol/L l-Glutamine. CWR-R1-D567 cells were maintained in RPMI1640 containing 10% charcoal-stripped serum and 2 mmol/L l-Glutamine. VCaP cells were maintained in DMEM (high glucose) containing 10% FBS, 2 mmol/L l-Glutamine, 2 mmol/L sodium pyruvate, and 2 mmol/L of nonessential amino acids solution (Sigma-Aldrich). All cell lines were authenticated by short tandem repeat profiling by CellBank Australia in 2017–2020 and were regularly screened for potential *Mycoplasma* contamination.

### Patient-derived Xenograft Explant Studies

Patient-derived xenografts (PDX) were established by the Melbourne Urological Research Alliance (MURAL). Written informed consent was obtained from all patients following Human Research Ethics Committee (Institutional Review Board) approvals from the Peter MacCallum Cancer Centre (11/102), Cabrini Health (03-14-04-08), and Monash University (1636, 12287). The studies were conducted in accordance with the National Statement on Ethical Conduct in Human Research produced by the National Health and Medical Research Council of Australia. All animal handling and procedures were approved by the Monash University Standing Committee of Ethics in Animal Experimentation (MARP 2012/158 and MARP/2014/085). CRPC PDXs 27.2 and 167.2 (19) were grown in an *ex vivo* culture system as described previously (20). Tumor fragments were treated for 48 hours with 10 or 100 nmol/L MeT, DHT or vehicle control. IHC of Ki67 and CC3 was performed as described previously (20). PDXs were routinely authenticated using short tandem repeat profiling (GenePrint 10, Promega) at the Australian Genome Research Facility.

### Cell Viability Assays

Cells were seeded at varying densities (depending on the doubling time of cell lines and length of the proliferation assay) in 6-well plates and incubated at 37°C and 5% CO_2_ for at least 24 hours to allow cells to be attached to the plate surface before treatment. At the appropriate timepoints, cells were treated with freshly prepared drugs (as indicated in figure legends), followed by incubation at 37°C and 5% CO_2_ until next timepoint. Treatments were refreshed every 2–3 days. At the end of each timepoint, cell viability was assessed using Trypan blue exclusion assays. The impact of MeT (1 and 100 nmol/L) on the proliferation of RM1 cells was assessed in a 96-well format using the sulforhodamine B-binding assay over 5 days with a seeding density of 500 cells per well, as described previously (21). Treatment commenced 24 hours postseeding. Endpoint absorbance was measured at 550 nm.

### Transactivation Assays

AR transactivation assays were performed in 96-well plates essentially as described previously (22). LNCaP cells were used to test the transactivation of endogenous AR, whereas PC-3 cells were used to test the transactivation of exogenous AR. Cells were transfected with 1 ng of pcDNA-AR (PC3 only) and 100 ng of a reporter construct containing three copies of the Probasin enhancer (pGL4.14-PB3-luc) using LipofectAMINE 2000 (GIBCO-BRL), according to the manufacturer's instructions. Following transfection, cells were treated for 24 hours in phenol red–free medium supplemented with the different doses of MeT and DHT, and luciferase activity was determined in cell lysates using the Luciferase Reporter Gene Assay Kit (Promega) and a plate reading luminometer (Top Count).

### Chromatin Immunoprecipitation sequencing

LNCaP cells were seeded at 5 × 10^6^ cells per plate in 15 cm plates in phenol red–free medium supplemented with 5% dextran-coated charcoal-stripped FBS and allowed to grow for 2 days prior to treatment with vehicle (ethanol), 1 nmol/L MeT or 1 nmol/L DHT for 4 hours. Subsequently, cells were fixed with formaldehyde and chromatin immunoprecipitation (ChIP) was performed essentially as described previously (23) using an Abcam AR antibody (ab108341; RRID: AB_10865716). For each treatment condition, two biological replicates were generated. After DNA quantification with Qubit dsDNA HS assay (Thermo Fisher Scientific), 5 ng of DNA (ChIP-enriched or input) was used for library preparation using a TruSeq ChIP Library Prep kit (Illumina). Sequencing was performed on an Illumina Nextseq 500 platform (single-end protocol, 75 bp read length) at the South Australian Genomics Centre (SAGC). Mapping and processing of fastq files were performed as described previously (24). Deeptools (25) was used to convert BAM files to bigwig and for visualizing ChIP sequencing (ChIP-seq) data as heatmaps. Consensus cistromes, comprised of peaks called in both replicates of each specific experimental condition, were created using BEDTools (26). Annotation of peaks with neighboring genes was performed using Cisgenome v2.0 (27). HOMER (28) was used to generate histograms of tag density around peaks (annotatePeaks.pl -size 6000 -hist 25) and also to identify known motifs enriched within peak sets (findMotifsGenome.pl -size 200). Alignments were visualized and interrogated using the Integrative Genomics Viewer v2.3.80 (29). ChIP-seq data have been deposited in the Gene Expression Omnibus (GEO; GSE187414).

### RNA Sequencing

LNCaP cells were seeded at a density of 2 × 10^5^ cells per well in 6-well plates, allowed to attach for 24 hours, then treated with vehicle, 1 nmol/L MeT or 1 nmol/L DHT. Total RNA was extracted at 6 and 24 hours after treatment using TRIzol. For each treatment condition, three biological replicates were generated. The integrity of RNA was first assessed using a 2100 Bioanalyzer system (Agilent). RNA concentration was quantified by Nanodrop 2000 (Thermo Fisher Scientific) and total RNA (2 μg) was supplied to the SAGC. RNA sequencing (RNA-seq) libraries were constructed using a TruSeq Total RNA HT kit (Illumina) and libraries were sequenced on the Illumina NextSeq 500 platform (stranded, paired-end 75 bp reads).

The quality of raw data was initially assessed using FastQC (http://www.bioinformatics.babraham.ac.uk/projects/fastqc/). Raw FASTQ files were then filtered for short sequences using Cutadapt v1.16.6 (30) with the following settings: minimum overlap length in adaptor options: 3, minimum length in filter options: 20, maximum error rate: 0.1, quality cutoff: 20. RNA-seq data have been deposited in the GEO (GSE187414).

To evaluate expression of protein-coding genes, reads were mapped against the human reference genome (hg19) using STAR version 2.6.0b-2 (31) with default parameters. FeatureCounts was used to count and assign the mapped reads to genomic features (32). Count tables generated by featureCounts were used for differential expression analysis using R version 3.2.3 and edgeR version 3.3 (33) as described previously (34). Heatmaps summarizing RNA-seq data were generated using ClustVis (35).

To evaluate expression of transposable elements (TE), reads were re-mapped against the human reference genome (hg19) using STAR version 2.6.0b-2 (31) with parameters that retained multiply mapped reads (–runThreadN 4 –outSAMtype BAM SortedByCoordinate –runMode alignReads –outFilterMultimapNmax 1000 –outFilterMismatchNmax 3 – outMultimapperOrder Random –winAnchorMultimapNmax 1000 –alignEndsType EndToEnd –alignIntronMax 1 –alignMatesGapMax 350). HOMER was used to count and assign the mapped reads to different classes of TEs (i.e., classes within the LTR, LINE, and SINE families). Count tables generated by HOMER were used to make a principal component analysis (PCA) plot with ClustVis.

### Gene Set Enrichment Analysis and Pathway Analysis

Gene set enrichment analysis (GSEA; Preranked analysis; ref. 36) was done as described previously (37). Identification of enriched pathways in gene sets was done using WebGestalt 2019 with a FDR cutoff of 0.01 (38).

### Apoptosis Assays

Cells were collected in FACS binding buffer (47 mL of Hanks' buffered saline buffered saline, 500 μL of HEPES solution, and 2.5 mL of 100 mmol/L CaCl_2_), staining with Annexin V PE (BD Biosciences) and 1 mmol/L 7-Aminoactiomycin D (Thermo Fisher Scientific). Apoptosis was measured by flow cytometry using a LSRFortessa X20 Cell Analyzer (BD Biosciences).

### Cell-cycle Analysis

LNCaP cells were seeded in 6-well plates and incubated overnight at 37°C and 5% CO_2_. Three days after treatment, cells were washed with a freshly prepared wash buffer containing PBS with 2% FBS, followed by trypsinization. The cell suspension was added to a 5 mL FACS tube containing cell culture media that had been collected earlier. Tubes were centrifuged at 700 × *g* for 5 minutes and cell pellets were resuspended and washed with 1mL PBS, followed by centrifugation at 700 × *g* for 5 minutes. After removing supernatants, cell pellets were resuspended in residual liquid by flicking the tubes. Subsequently, 1 mL ice-cold 70% ethanol in PBS was added into tubes containing the cell suspensions and fixed overnight at 4°C. Following cell fixation, cells were centrifuged at 700 × *g* for 5 minutes and the cell pellets were washed twice with 1 mL Hanks’ Balanced Salt Solution + 2% FBS. Cells were then stained with 1 mL of 4´-6-Diamidino-2-phenylindole (DAPI; 10 μg/mL). The prepared cell suspension was used for cell-cycle analysis based on DNA content using a FACSCanto II flow cytometer (BD Biosciences); analysis was carried out using FlowJo software.

### qRT-PCR Analysis of mRNA from Human Cells

Total RNA from human cell lines was extracted using TRI Reagent (Sigma), according to the manufacturer's instructions. Total RNA was treated with Turbo DNA-free kit (Invitrogen), and reverse transcribed using iScript Reverse Transcriptase Supermix kit (Bio-Rad). PCR was done in triplicate using a CFX384TM Real-Time System, as described previously (39). Levels of *GAPDH* were used for normalization of qRT-PCR data. Primer sequences are listed in Supplementary Table S1.

### qRT-PCR Analysis of mRNA from Mouse Cells

mRNA was extracted using the Qiagen Rneasy Plus Mini Kit (Qiagen) according to the manufacturer's instructions and reverse transcribed using iScript Reverse Transcriptase Supermix cDNA for qRT-PCR kit (Bio-Rad). qRT-PCR was performed using PowerUp SYBR Green Master Mix (Applied Biosystems) to quantify gene expression on the CFX384TM Real-Time System (Bio-Rad) as described previously (18). Gene expression (arbitrary units) was calculated as mean relative transcript abundance by methods outlined previously (40) and expressed relative to a housekeeping gene, *Hprt*. Primer sequences are listed in Supplementary Table S1.

### Western Blotting

Protein extraction from cells using RIPA buffer (human cell lines) or hypotonic lysis buffer (RM1) and Western blotting was done essentially as described previously (39). Primary antibodies used in human Western blotting were: TBK1 (Cell Signaling Technology 3013; RRID: AB_2199749); phospho-Ser172-TBK1 (Cell Signaling Technology D52C2; RRID: AB_10693472); RIG-I (Santa Cruz Biotechnology SC-376845; RRID: AB_2732794); and GAPDH (Millipore MAB374; RRID: AB_347661). Primary antibodies used in murine Western blotting were: AR (Abcam ab108341; RRID: AB_10865716); and GAPDH XP (Cell Signaling Technology D16h11; RRID: AB_10622025). Horseradish peroxidase–conjugated anti-rabbit and anti-mouse IgG secondary antibodies (Dako) were used and immunoreactive bands visualized using Clarity Western ECL Substrate (Bio-Rad).

### Immunofluorescence

LNCaP cells were seeded on glass coverslips in 6-well plates. To improve cell adhesion, glass coverslips were coated with 1:8 diluted L-Poly-Lysine. After treatment, cells were fixed in 4% paraformaldehyde for 10 minutes, permeabilized in 0.1% Triton X-100 for 15 minutes, and blocked in 2.5% BSA (for phospho-Histone H2A.X) or 5% BSA (for J2) solution for 1 hour. The coverslips then were incubated with anti-γH2AX primary antibody (Millipore 05-636; RRID: AB_309864) or J2 antibody (SCICONS 10010200; RRID: AB_2651015; both used at 1:1,000) overnight at 4°C, followed by washing (twice with 5-minute intervals) and then incubation with a fluorescent-tagged secondary antibody for 1 hour at room temperature. Cell nuclei were visualized by costaining the cells with DAPI (Invitrogen) for 1 minute. Imaging was carried out using a confocal microscope (Olympus FV3000 Confocal Microscope). To quantify the number of γH2AX foci per nucleus, images were analyzed using Image J software: (i) the number of cells (i.e., DAPI-stained nuclei) were counted in each image by Analyze Particles tool; (ii) the number of γH2AX foci in each image was quantified using the Find Maxima tool, which was performed using the noise tolerance parameter adjusted for positive control; (iii) the average number of foci per nucleus for each treatment was calculated by counting γH2AX foci from 70 to 150 cells per treatment across multiple microscope fields. To quantify J2 signal, Image J (41) was used to measure signal intensity at 4–5 regions of interest (ROI) per treatment; total signal intensity was then normalized to the cell count within each ROI.

### Quantification of LINE-1 DNA Methylation

Cells were grown and treated in 6-well plates and genomic DNA was isolated using QIAamp DNA Mini kits, according to the manufacturer's instructions. To quantify the DNA methylation in DNA samples, Global DNA Methylation-LINE-1 Kits (Active Motif) were used to assess the methylation of 5-mC status at long interspersed nucleotide element 1 (LINE-1) elements, as specified by the manufacturer.

### Intracellular Cytokine Staining for T‐cell Specificity

For assessment of androgen effects on antigen presentation in cancer cells, RM1 cells were treated with MeT, DHT or vehicle control. Following 72 hours, RM1 cells (5 × 10^4^) were cocultured with *in vitro* expanded RM1‐specific CD8^+^ T cells for 5 hours in the presence of 10 μg/mL Brefeldin A. Intracellular cytokine staining (ICS) assays for production of IFNγ were carried out as described previously (18).

### Analysis of Prostate Cancer Clinical Transcriptomic Data

Clinical transcriptomic datasets [The Cancer Genome Atlas (TCGA; ref. 42) and SU2C (43)] were downloaded from cBioportal (44). The activity of AR signaling and other pathways (i.e., antiviral mechanism by IFN-stimulated genes, MHC class I antigen processing and presentation) in these datasets was estimated by single-sample GSEA (ssGSEA; ref. 45); ssGSEA was implemented using the Broad Institute's public GenePattern server, using rank normalization and default parameters.

### Statistical Analysis

Statistical analyses for grouped quantitative data were carried out using two-tailed unpaired *t* test or ANOVA (GraphPad Prism 9). The relationships between activity scores were determined using Pearson correlation coefficient (Graphpad Prism 9). Further details of statistical tests are provided in the figure legends. Statistical significance was defined as *P* < 0.05.

### Data Availability Statement

The data generated in this study are publicly available in GEO at GSE187414.

## Results

### MeT is a Potent Stimulator of AR Activity and Suppressor of Prostate Cancer Cell Growth

**FIGURE 1:**
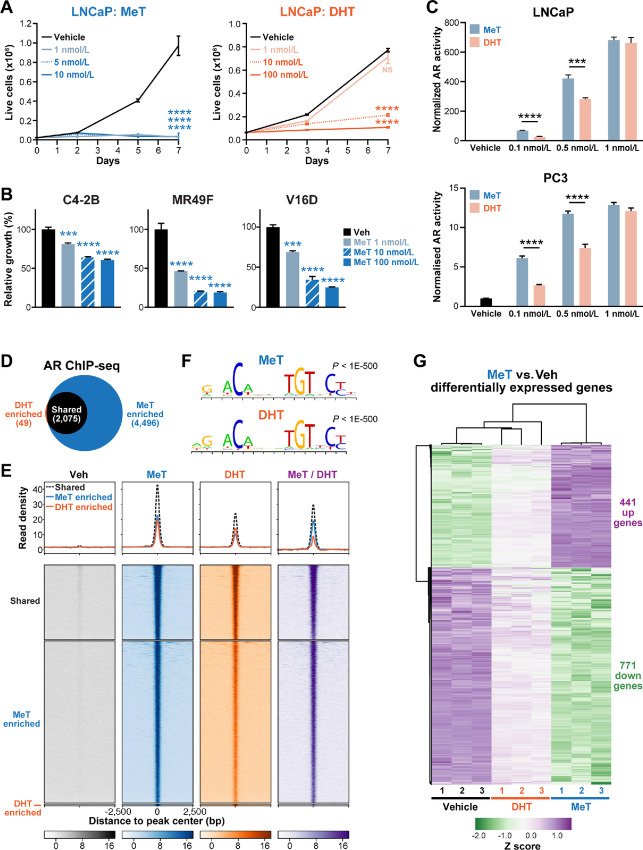
MeT has potent androgenic and growth suppressive activity in prostate cancer cells. **A,** MeT potently suppresses growth of LNCaP cells (left graph), as determined by Trypan blue growth assay. The response of cells to DHT is shown on the right. Error bars are ± SEM. *P* values (using day 7 data) were determined using ANOVA and Dunnett multiple comparisons tests (*, *P* < 0.05; **, *P* < 0.01; ***, *P* < 0.001; ****, *P* < 0.0001). NS, not significant. **B,** MeT inhibits the growth of CRPC models of prostate cancer (C4-2B, MR49F, and V16D), as determined by Trypan blue growth assay. Statistical analysis was as for **A**. **C,** Activation of AR transcriptional activity by MeT in LNCaP cells (top) and PC3 cells (bottom). PC-3 cells were transfected with plasmids expressing AR and a probasin-luciferase reporter for 4 hours prior to 20 hours treatment with MeT or DHT; LNCaP cells were transfected only with the probasin-luciferase reporter. Transcriptional activity values represent the mean of six technical replicates; results are representative of three independent experiments. Error bars are SEM. Unpaired *t* tests were used to compare MeT and DHT (***, *P* < 0.001; ****, *P* < 0.0001). **D,** Venn diagram showing the overlap of AR cistromes in LNCaP cells treated with DHT or MeT (1 nmol/L each). **E,** Read density plots (top) and heatmaps (bottom) representing AR ChIP-seq peak sets from **D**. Far right heatmaps (“MeT/DHT”) represent MeT ChIP-seq signals corrected to DHT ChIP-seq signals. **F,** Most highly enriched motifs in the MeT (top) and DHT (bottom) cistromes. Motifs were identified using a *de novo* Gibbs motif sampling approach. *P* values represent enrichment over genomic background, calculated using Fisher exact tests. **G,** Heatmap of RNA-seq data for genes differentially expressed by 24 hours of MeT treatment (compared with Vehicle; FDR < 0.05). The heatmap was generated using ClustVis (35) after applying unit variance scaling to each gene.

To investigate the response of prostate cancer cells to MeT, we first examined changes in cell growth. MeT has growth-inhibitory activity in LNCaP cells grown in normal maintenance culture media (i.e., androgen replete conditions) at doses as low as 1 nmol/L (Fig. 1A). Conversely, DHT only suppressed cell growth at doses >1 nmol/L in this model (Fig. 1A). Western blotting demonstrated that both androgens stabilized AR protein levels, as expected (Supplementary Fig. S1A). Growth of the CRPC cell lines C4-2B, MR49F, and 22Rv1 was inhibited by MeT and DHT at doses ranging from 1 to 100 nmol/L (Fig. 1B; Supplementary Fig. S1B). MeT also exhibited antitumor activity against two PDXs derived from patients with CRPC (19) in an *ex vivo* culture system (Supplementary Fig. S1C). Neither the AR-negative model PC3 nor the R1-D567 model, which expresses an AR variant that lacks the ligand-binding domain (ARv567es), was affected by MeT (Supplementary Fig. S1D), indicating that growth suppression was a consequence of binding of MeT to full-length AR.

To better understand the activity of MeT in prostate cancer cells, we undertook a series of molecular assays. First, we compared MeT and DHT in a classic transcriptional activation assay using a probasin promoter:luciferase reporter construct (PB3-luc (46)). At low concentrations (0.1–0.5 nmol/L), MeT more potently activated endogenous AR in LNCaP cells and exogenously supplied AR in PC3 cells (Fig. 1C). Subsequently, to evaluate MeT activation of AR signaling at a global level and in a more physiologic setting, we conducted AR ChIP-seq in LNCaP cells. Consensus AR chromatin-binding datasets (cistromes) were generated from two replicates for each treatment condition (see Materials and Methods). The AR-MeT cistrome was approximately 3-fold larger than the equivalent AR-DHT cistrome, with almost all AR-DHT binding sites shared with AR-MeT binding sites (Fig. 1D and E; Supplementary Dataset S1). Moreover, at the set of sites shared between the two conditions (i.e., MeT and DHT), MeT elicited substantially stronger AR binding to chromatin (Fig. 1E, far right heatmap). Despite the stronger ChIP-seq signal of AR-MeT compared with AR-DHT, there was clear evidence of DHT-induced binding of AR to many sites classified as being specific to MeT; hence, we refer to these as “MeT-enriched” rather than “MeT-specific” AR binding sites (Fig. 1E). The high degree of similarity between the AR-MeT and AR-DHT cistromes suggested that, in general, MeT did not lead to new AR-binding events but rather enhanced its interaction with canonical regulatory elements. This concept was supported by motif analysis of the MeT-enriched, DHT-enriched, and shared peak sets, all of which were highly enriched for androgen response elements and Forkhead box motifs (Fig. 1F; Supplementary Dataset S1).

The hypothesis that MeT exerts largely quantitative, rather than qualitative, differences to AR activity was further reinforced by transcriptomic analysis (RNA-seq), which revealed that genes differentially expressed in response to MeT (*n* = 1,212, FDR ≤ 0.05) were also altered by DHT in a directionally consistent manner, albeit to a lesser degree (Fig. 1G; Supplementary Dataset S2). This effect was most striking when assessing the 285 genes that were differentially expressed by DHT compared with vehicle (FDR ≤ 0.05): 99% (282/285) of these genes were also regulated by MeT, all of those 282 genes were altered in the same direction by both hormones, and 99% (281/282) were more strongly regulated by MeT than by DHT (average difference in log fold change: downregulated = 1.51, upregulated = 1.17; Supplementary Dataset S2). The majority of genes altered by either hormone were downregulated (Fig. 1G; Supplementary Dataset S2). Gene set analysis revealed that pathways altered by MeT and DHT were also highly concordant, with both hormones impacting on cell cycle, DNA replication, and DNA repair processes (Supplementary Dataset S2). Collectively, these findings suggest that MeT is a potent activator of AR signaling that increases the magnitude, but not the spectrum, of the canonical DHT-regulated gene expression program.

### MeT Suppresses DNA Replication and Repair Pathways in Prostate Cancer Cells

**FIGURE 2:**
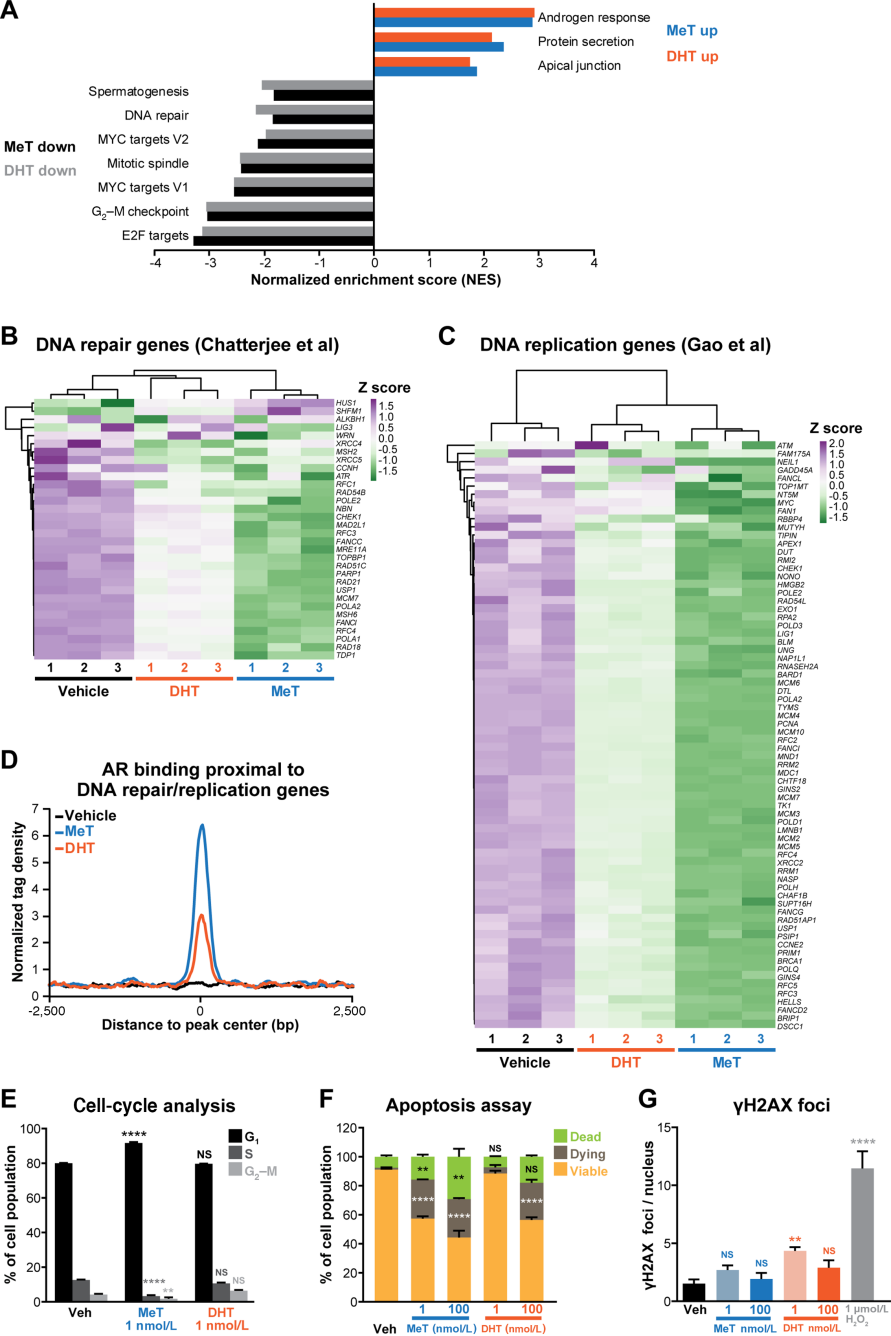
DNA replication and repair pathways are repressed by potent androgenic stimulation of prostate cancer cells. **A,** Normalized enrichment scores (NES) for Hallmark gene sets (47) representing RNA-seq data from LNCaP cells treated with 1 nmol/L MeT or DHT for 24 hours. **B** and **C,** Heatmap of RNA-seq data for androgen-regulated genes associated with DNA repair (48) and DNA replication (49) in LNCaP cells treated with 1 nmol/L MeT or 1 nmol/L DHT for 24 hours. Heatmaps were generated using ClustVis (35) after applying unit variance scaling to each gene. **D,** Average read density plots for AR chromatin binding proximal (<100 kb) to DNA repair/replication genes in LNCaP cells treated with 1 nmol/L MeT or 1 nmol/L DHT for 4 hours. **E,** Cell-cycle analysis by DAPI labeling and flow cytometry after 72 hours of treatment with 1 nmol/L MeT or 1 nmol/L DHT. Unpaired *t* tests were used to compare data at different cell-cycle phases (i.e., G_1_, S, and G_2_–M) between treatment groups (**, *P* < 0.01; ****, *P* < 0.0001). **F,** Flow cytometry–based Annexin V/7-AAD apoptosis assays after 96 hours of treatment with MeT or DHT. Data represent the mean ± SEM of triplicate samples and are representative of three independent experiments. Dead and dying cell proportions were compared with vehicle using ANOVA and Tukey multiple comparison tests (**, *P* < 0.01; ****, *P* < 0.0001; NS, not significant). **G,** Assessment of DNA DSBs after androgen treatment. γH2AX foci were quantitated in LNCaP cells 6 hours after treatment with indicated doses of MeT, DHT, or a positive control (H_2_O_2_). Error bars are SEM. *P* values (day 7) were determined using ANOVA and Dunnett multiple comparisons tests (*, *P* < 0.05; **, *P* < 0.01; ***, *P* < 0.001; ****, *P* < 0.0001; NS, not significant).

Given its potent growth-inhibitory activity, we hypothesized that further dissecting the MeT-regulated transcriptome of MeT could yield new mechanistic insights into AR's tumor suppressive activity in prostate cancer. GSEA (36) was used to identify “Hallmark” gene sets (47) altered by treatment with this potent androgen. The most upregulated hallmark gene sets for both MeT and DHT were “androgen response,” “protein secretion.” and “apical junction” (Fig. 2A), providing further evidence that MeT regulates a transcriptional program that is highly similar to physiologic androgens. Hallmarks that were robustly repressed by MeT/DHT were related to DNA replication and repair (i.e., E2F targets, MYC targets, G_2_–M checkpoint, mitotic spindle, DNA repair; Fig. 2A), analogous to what has been reported for high-dose androgen treatment previously (48–50). When we examined curated DNA repair (48) and DNA replication (49) gene sets reported to be repressed by high-dose androgen treatment, we observed that MeT downregulated these to a considerably greater extent than DHT (Fig. 2B and C). Many of these genes have been purported to be directly regulated by AR on the basis of its binding to proximal regulatory elements (49). Indeed, we found that AR binding near these genes was strongly stimulated by MeT and, to a lesser extent, DHT (Fig. 2D).

A reported consequence of suppression of DNA repair and replication pathways by high-dose androgen treatment is cell-cycle arrest (48, 51). Flow cytometry revealed that MeT caused accumulation of cells in G_1_-phase and consequent reduction of cells in S- and G_2_–M-phases (Fig. 2E). The same dose of DHT did not have a significant effect on the cell cycle (Fig. 2E), providing additional evidence that MeT is a more potent androgen in terms of prostate cancer cell growth suppression. Cell-cycle arrest caused by MeT (Fig. 2E) did not fully explain its potent antiproliferative activity in cell growth assays (Fig. 1A). Flow cytometric analysis of Annexin V and 7-AAD assays revealed that MeT and DHT caused LNCaP cells to undergo apoptosis (Fig. 2F), demonstrating that these androgens exert both cytostatic and cytotoxic effects. The lower dose of MeT (1 nmol/L) had a significantly stronger pro-apoptotic effect than 1 nmol/L DHT (one-way ANOVA with Tukey multiple comparisons test: dead cells, *P* = 0.003; dying cells, *P* < 0.0001).

A proposed mediator of G_1_ arrest by high-dose androgen treatment is increased DNA damage, occurring via a combination of AR-mediated double-stranded breaks (DSB; ref. 52) and downregulation of DNA repair genes (48). However, MeT did not significantly increase the number of γH2AX foci (Fig. 2G; Supplementary Fig. S1E), a marker of DSBs, suggesting that DNA damage is not a major mechanism underlying its growth-suppressive activity in prostate cancer cells. Low dose, but not high dose, DHT caused a minor increase in the number of γH2AX foci (Fig. 2G; Supplementary Fig. S1E), potentially representing a differential mode of action between the two androgens in relation to DNA damage and repair.

### MeT Causes DNA Hypomethylation in Prostate Cancer Cells

**FIGURE 3:**
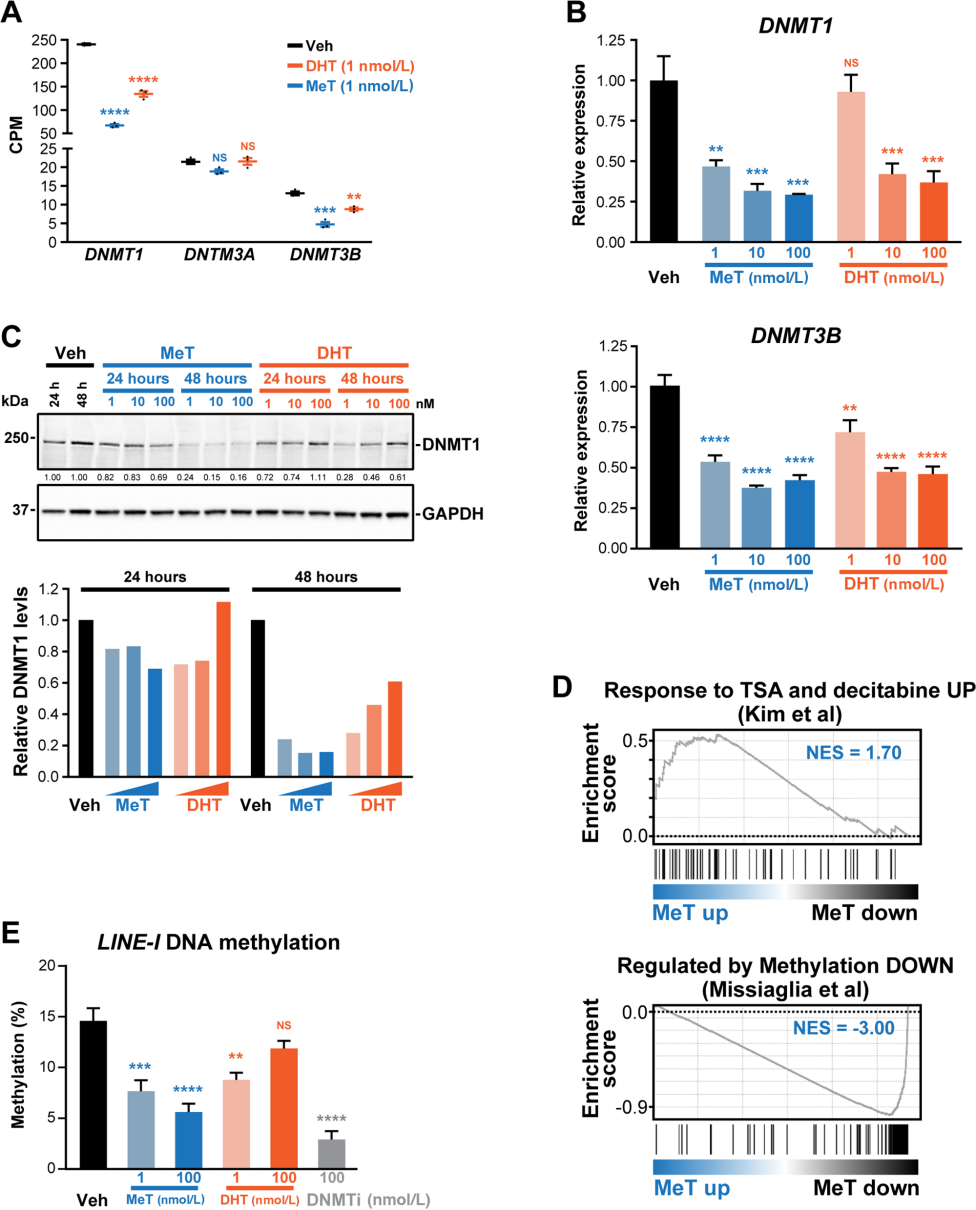
MeT downregulates DNA methyltransferases and causes DNA hypomethylation. **A,** Expression of *DNMT1*, *DNTM3A,* and *DNMT3B*, as determined by RNA-seq, in LNCaP cells following 24 hours of treatment with MeT or DHT (1 nmol/L each) or a vehicle control. CPM, counts per million reads. Middle line, mean; above and below, ± SEM. *P* values (treatment compared with vehicle) were determined using ANOVA and Dunnett multiple comparisons tests (*, *P* < 0.05; **, *P* < 0.01; ***, *P* < 0.001; ****, *P* < 0.0001). **B,** Expression of *DNMT1* (top) and *DNMT3B* (bottom), as determined by qRT-PCR, following 24 hours of treatment with MeT or DHT (1 nmol/L each) or a vehicle control. Gene expression was normalized to *GAPDH*; expression for Vehicle was set to 1. Error bars are SEM; *P* values (treatment compared with vehicle) were determined using ANOVA and Dunnett multiple comparisons tests (*, *P* < 0.05; **, *P* < 0.01; ***, *P* < 0.001; ****, *P* < 0.0001). **C,** Representative Western blot analysis showing DNMT1 protein levels following treatment of LNCaP cells with the indicated doses of MeT or DHT or vehicle control for 24 and 48 hours. GAPDH is shown as a loading control. Quantification of DNMT1 protein (normalized to GAPDH, Veh set to 1) is shown as values below the immunoblot. **D,** Association between MeT-induced genes and a gene set upregulated by DMNT inhibitors TSA and decitabine (top) (54) and between MeT-repressed genes and a set of genes downregulated following treatment with decitabine (bottom; ref. 55), as demonstrated by GSEA. NES, normalized enrichment score. **E,** Global DNA methylation (5 mC; % methylation of *LINE-1* elements) in LNCaP cells treated with indicated doses of MeT or DHT for 6 days. Decitabine (“DNMTi”, 100 nmol/L) was used as a positive control. *P* values (treatment compared with vehicle) were determined using ANOVA and Dunnett multiple comparisons tests (*, *P* < 0.05; **, *P* < 0.01; ***, *P* < 0.001; ****, *P* < 0.0001).

AR has a major role in regulating the epigenome via interplay with and transcriptional regulation of chromatin remodeling factors (53), although little is known about how these mechanisms are altered in the context of high-dose androgen treatment. Our RNA-seq data revealed that MeT strongly downregulated the DNA methyltransferases *DNMT1* and *DNMT3b* in LNCaP cells (Fig. 3A), which we validated by qRT-PCR (Fig. 3B) and Western blotting in multiple cell lines (Fig. 3C; Supplementary Fig. S2). Gene signatures of response to DNMT inhibitors (54, 55) were altered by MeT (Fig. 3D), suggesting that DNA hypomethylation and subsequent effects on transcription were occurring downstream of DNMT downregulation. To directly test this idea, we assayed for 5-Methylcytosine at LINEs, a proxy for global DNA methylation. In support of our expression profiling data, a decrease in global DNA methylation levels was observed in response to MeT and, to a lesser extent, DHT, after 6 days of treatment (Fig. 3E).

### MeT Induces Transcription of Transposable Elements and Causes Accumulation of double-stranded RNA

**FIGURE 4:**
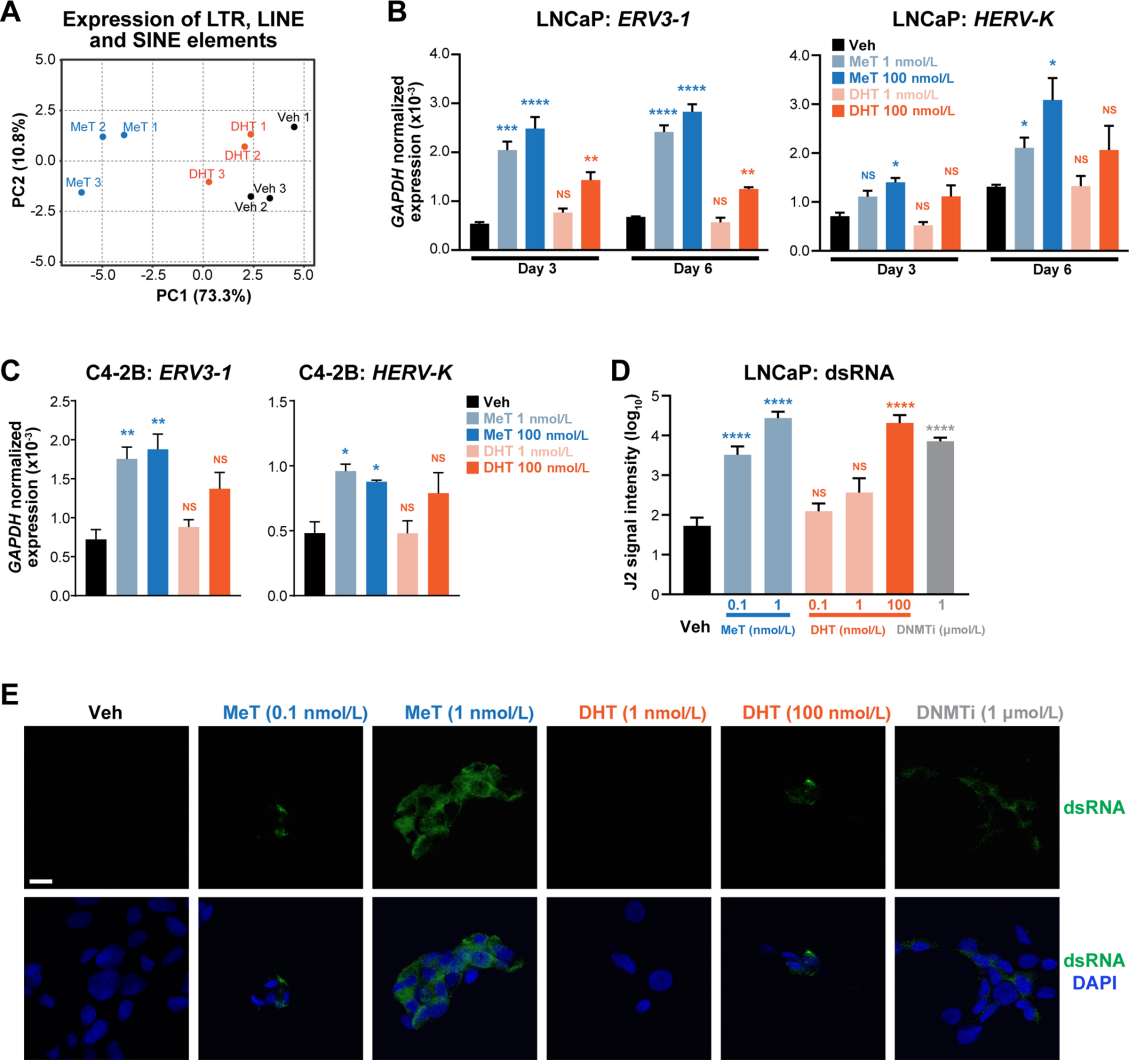
Induction of transposable element expression by MeT is associated with production of dsRNA. **A,** PCA of transposable element expression (long terminal repeats, LINE and SINE elements) from RNA-seq data following treatment of LNCaP cells with MeT or DHT (1 nmol/L each) for 24 hours. The plot was generated using ClustVis (35) after applying unit variance scaling to each element. **B,** Expression of *ERV3-1* and *HERV-K*, as determined by qRT-PCR, following 3 or 6 days of treatment of LNCaP cells with indicated doses of MeT, DHT, or a vehicle control. Expression of ERVs was normalized to *GAPDH*. Error bars are SEM; *P* values (treatment compared with vehicle) were determined using ANOVA and Dunnett multiple comparisons tests (*, *P* < 0.05; **, *P* < 0.01; ***, *P* < 0.001; ****, *P* < 0.0001). **C,** Expression of *ERV3-1* and *HERV-K*, as determined by qRT-PCR, following 3 days of treatment of C4-2B cells with indicated doses of MeT, DHT, or a vehicle control. Expression of ERVs was normalized to *GAPDH*. Error bars are SEM; statistical testing was as in **B**. **D,** Quantitation of cellular dsRNA in LNCaP cells by immunofluorescent staining with J2 mAb following 3 days of treatment with MeT, DHT, or a DNMTi, decitabine. Error bars are SEM; *P* values (treatment compared with vehicle) were determined using ANOVA and Dunnett multiple comparisons tests (****, *P* < 0.0001). **E,** Representative images of J2 immunofluorescence. J2 signal, representing cellular dsRNA, is in green. Nuclei were counterstained with DAPI (blue). Scale bar, 20 μmol/L.

The transcription of TEs, which constitute approximately 45% of the human genome (56) and are comprised of distinct families including endogenous retroviruses (ERV), LINEs, and short interspersed nuclear elements (SINE), is heavily influenced by DNA methylation (57). Thus, we hypothesized that loss of DNA methylation in response to MeT could lead to altered TE expression. To test this hypothesis, we first interrogated levels of different TE classes within the ERV/LINE/SINE families in our short-term (24 hour) RNA-seq data. Similar to our analyses of the coding transcriptome, MeT caused substantial changes to expression of TEs whereas DHT had a less pronounced effect (Fig. 4A).

Having established that potent androgen stimulation could alter the expression of TEs in 24 hours, we measured specific transcripts after 3–6 days of treatment, based on the earlier observation that loss of DNA methylation occurred over an equivalent period (Fig. 3E). We initially focused our attention on LINEs, because these elements were specifically evaluated in the DNA methylation assays. *LINE-1* was weakly induced by MeT after 6 days of treatment, but its expression was not altered by DHT treatment (Supplementary Fig. S3A), a finding that was recapitulated in the CRPC cell line C4-2B (Supplementary Fig. S3B). Subsequently, we measured expression of the major family members of ERVs, because these sequences of viral origin are known to influence various biological processes in cancer cells, including innate immune responses (58). MeT induced *ERV3-1* and *HERV-K* transcripts in LNCaP cells (Fig. 4B); *HERV-E* and *HERV-W* were not significantly altered but exhibited a trend toward upregulation (Supplementary Fig. S3C). Analogous results—significant induction of *ERV3-1* and *HERV-K* but not *HERV-E* or *HERV-W*—were observed in the C4-2B model, demonstrating that this response occurs in both androgen-dependent (LNCaP) and androgen-independent (C4-2B) models of prostate cancer (Fig. 4C; Supplementary Fig. S3D). As for protein-coding transcripts, equivalent doses of DHT caused similar qualitative changes to LINE/ERV expression but quantitatively weaker effects (Fig. 4B and C; Supplementary Fig. S3). Collectively, these findings demonstrate that the potent synthetic androgen MeT can induce expression of transposable elements, including ERVs, in a context-dependent manner in prostate cancer cells.

Expression of some ERVs occurs bidirectionally and can thereby result in generation of double-stranded RNA (dsRNA; ref. 59). Because potent androgen treatment led to increased levels of the major classes of ERVs over a period of 3–6 days, we speculated that this could cause accumulation of dsRNA. Using an immunofluorescent approach with a dsRNA-specific antibody (J2), we found that MeT treatment elicited a significant increase in the level of cellular dsRNA in LNCaP cells (Fig. 4D and E). Indeed, 1 nmol/L MeT resulted in more detectable dsRNA than 100 nmol/L DHT and 1 μmol/L of decitabine, a DNMT inhibitor (DNMTi) previously reported to induce dsRNA in other cancer cell types (refs. 59–61; Fig. 4D and E). dsRNA levels were elevated in response to androgen treatment in another AR-positive model, VCaP (Supplementary Fig. S3E); the effect was less pronounced in this cell line compared to LNCaP, potentially due to higher background immunofluorescence levels. Collectively, these findings reveal that potent androgenic stimulation of prostate cancer cells leads to dysregulation of TE transcription that is associated with accumulation of ERV transcripts and dsRNA.

### MeT Activates IFN Signaling

**FIGURE 5:**
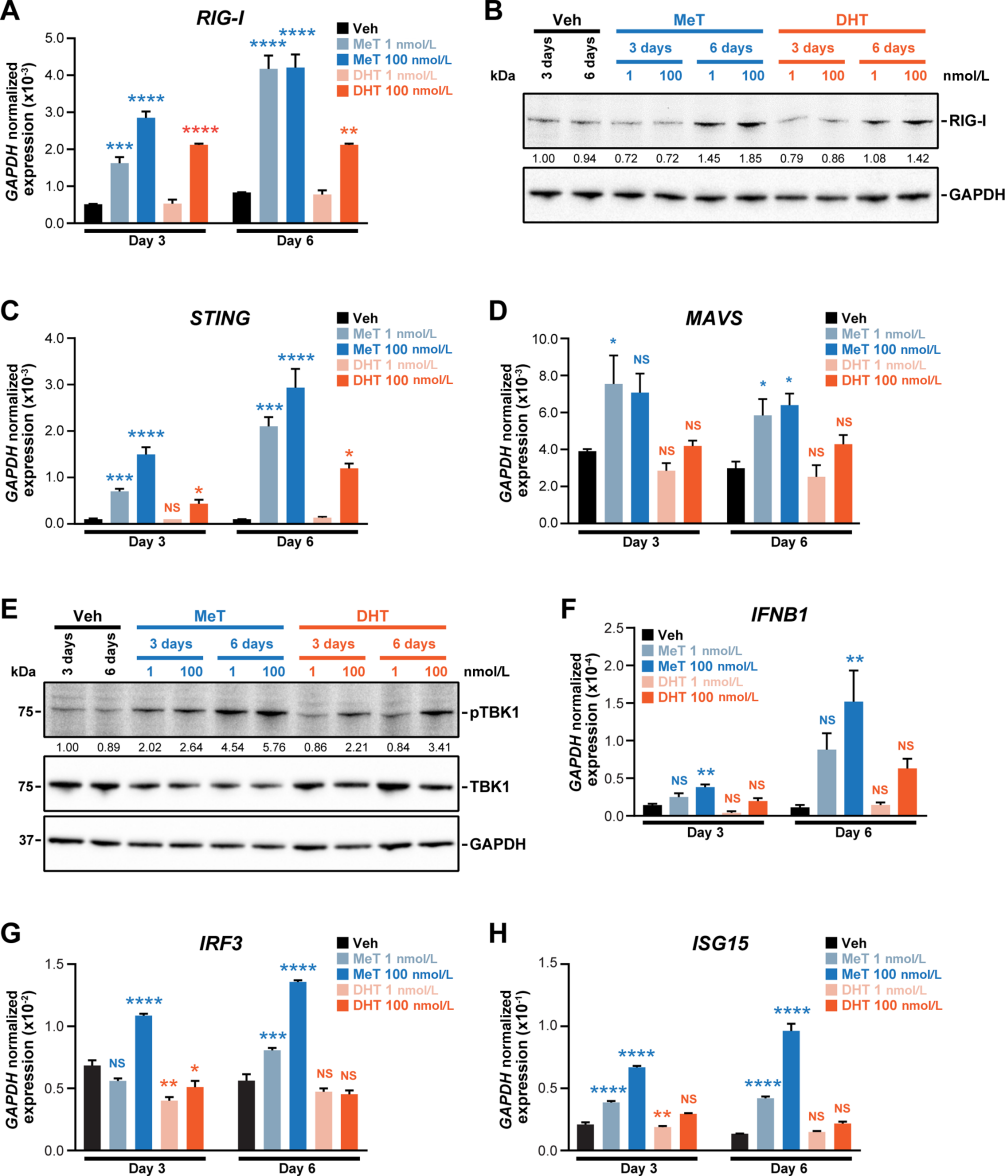
MeT activates an IFN-mediated antiviral response. **A,** Expression of *RIG-I* in LNCaP cells as determined by qRT-PCR following 3 or 6 days of treatment with the indicated doses of MeT or DHT or a vehicle control. Gene expression was normalized to *GAPDH*. Error bars are SEM; *P* values (treatment compared with vehicle at each timepoint) were determined using ANOVA and Dunnett multiple comparisons tests (**, *P* < 0.01; ***, *P* < 0.001; ****, *P* < 0.0001). **B,** Western blot analysis showing RIG-I protein levels following treatment of LNCaP cells with the indicated doses of MeT or DHT or vehicle control for 3 or 6 days. GAPDH is shown as a loading control. Expression of *STING* (**C**) and *MAVS* (**D**) in LNCaP cells as determined by qRT-PCR following 3 or 6 days of treatment with the indicated doses of MeT or DHT or a vehicle control. Normalization and statistical tests were as in **A** (*, *P* < 0.05; ***, *P* < 0.001; ****, *P* < 0.0001). **E,** Western blot analysis showing levels of total and phosphorylated TBK1 in C4-2B cells following treatment with the indicated doses of MeT or DHT or vehicle control for 3 or 6 days. GAPDH is shown as a loading control. Expression of *IFNB1* (**F**), *IRF3* (**G**), and *ISG15* (**H**) in LNCaP cells as determined by qRT-PCR following 3 or 6 days of treatment with the indicated doses of MeT or DHT or a vehicle control. Normalization and statistical tests were as in **A** (*, *P* < 0.05; **, *P* < 0.01; ***, *P* < 0.001; ****, *P* < 0.0001).

Induction of ERV transcription and accumulation of dsRNA can activate cellular responses similar to those elicited by infection with an exogenous virus, a phenomenon termed “viral mimicry” (58). Given the ability of MeT to modulate ERV transcription and induce dsRNA, we speculated that it could cause a viral mimicry response. To test this hypothesis, we first measured mRNA levels of the cytosolic pattern recognition receptor (PRR) RIG-I (encoded by the *DDX58* gene), which is a major sensor of dsRNA produced during viral infection. We observed induction of *RIG-I* in response to MeT and, to a lesser extent, DHT (Fig. 5A), which was confirmed by Western blotting (Fig. 5B). Another PRR involved in antiviral responses, STING, is best known for its role in sensing of cytosolic DNA but also serves as a detector of RNA viruses and can interact with RIG-I (62): similarly to RIG-I, STING was strongly upregulated by MeT in prostate cancer cells (Fig. 5C). Downstream of PRRs, the mitochondrial antiviral signaling protein (MAVS) and TANK Binding Kinase 1 (TBK-1) are required to activate innate immune antiviral responses (63). As expected, MeT treatment increased the levels of *MAVS* mRNA and phosphorylated (active) TBK-1; DHT again caused analogous but blunted responses (Fig. 5D and E). Sensing of dsRNA by PRRs leads to activation of type I IFN signaling (64). MeT treatment caused induction of *IRF3*, a transcription factor that can activate IFN expression (Fig. 5F), IFNβ (encoded by *IFNB1*; Fig. 5G) and *ISG15* (IFN-stimulated gene 15; Fig. 5H), an IFN-induced factor that plays an important role in the host antiviral response. Activation of IFN signaling by MeT was also observed in the CRPC model C4-2B (Supplementary Fig. S4). Collectively, these findings reveal that MeT activates an antiviral response, likely due to its ability to increase cellular levels of dsRNA.

### Association Between AR Activity and Antiviral Responses in Clinical Prostate Cancer

**FIGURE 6:**
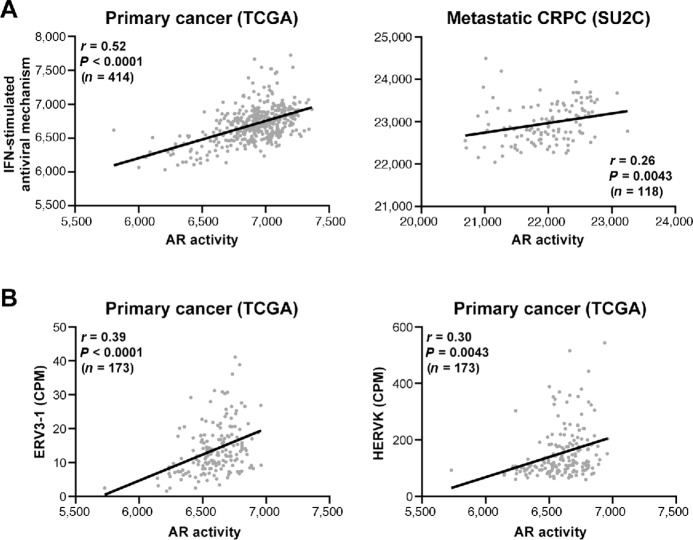
Positive association between AR signaling and antiviral responses in patient tumors. **A,** AR activity, based on a 267-gene signature (66), is associated with the Reactome “antiviral mechanism by IFN-stimulated genes” gene set in TCGA (left) and SU2C (right) datasets. Activity scores were calculated using ssGSEA. *P* and *r* values were determined using Pearson correlation tests. **B,** AR activity is associated with the levels of ERV3-1 (left) and HERVK in TCGA dataset. AR activity scores were calculated using ssGSEA. Counts per million (CPM) reads for ERV3-1 and HERVK (sum of all HERVK transcripts) were obtained from a published study (65). *P* and *r* values were determined using Pearson correlation tests.

Our mechanistic investigations using prostate cancer cell lines suggested that potent androgen stimulation could activate a viral mimicry response involving IFN signaling. To gain evidence for this concept in a more clinically relevant setting, we analyzed large transcriptomic datasets from patients with primary prostate cancer (TCGA) and metastatic CRPC (SU2C). Supporting our preclinical mechanistic work, we found a significant positive correlation between AR activity (66) and Reactome's “antiviral mechanism by IFN-stimulated genes” gene set (Fig. 6A). Moreover, by exploiting a study in which ERVs were quantitated in a subset of TCGA samples (65), we discovered a strong positive correlation between AR activity and the expression of ERVs in the ERV3-1 and HERV-K classes (Fig. 6B). No association between AR signaling and HERV-E (*r* = 0.079, *P* = 0.30) or HERV-W (*r* = −0.047, *P* = 0.54) classes was observed, corroborating our earlier findings that these classes of ERVs were not robustly induced by high-dose androgen treatment (Supplementary Fig. S3).

### MeT Can Enhance the Interaction Between Prostate Cancer Cells and T Cells

**FIGURE 7:**
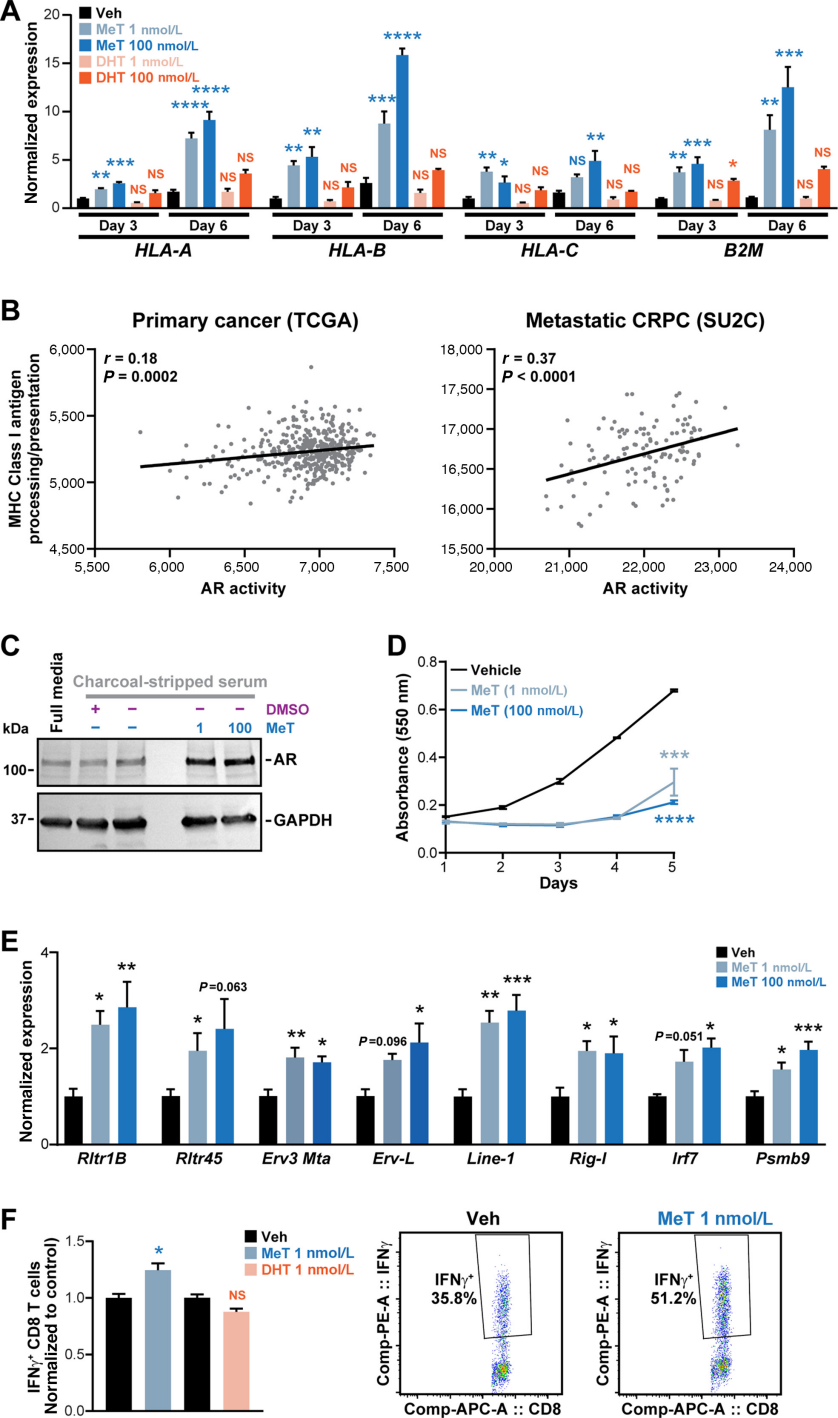
MeT elicits viral mimicry and enhances interplay with T cells in a mouse model of prostate cancer. **A,** Expression of *HLA* genes and *B2M*, as determined by qRT-PCR, following 3 or 6 days of treatment of LNCaP cells with the indicated doses of MeT or DHT or a vehicle control. Gene expression was normalized to *GAPDH*. Error bars are SEM; *P* values (treatment compared with vehicle at each timepoint) were determined using ANOVA and Dunnett multiple comparisons tests (*, *P* < 0.05; **, *P* < 0.01; ***, *P* < 0.001; ****, *P* < 0.0001). **B,** AR activity is associated with the Reactome “class I MHC-mediated antigen processing and presentation” gene set in TCGA (left) and SU2C (right) datasets. Activity scores were calculated using ssGSEA. *P* and *r* values were determined using Pearson correlation tests. **C,** Western blot analysis showing AR protein expression in RM1 cells following treatment with the indicated doses of MeT or vehicle control in both full (androgen replete) and charcoal-stripped (androgen depleted) media. GAPDH is shown as a loading control. **D,** MeT suppresses growth of RM1 cells, as determined by sulforhodamine B colorimetric assay mean absorbance (550 nm) is shown at the indicated timepoints; error bars are ± SEM. *P* values were determined using unpaired *t* tests at day 5 (***, *P* < 0.001; ****, *P* < 0.0001). **E,** Expression of ERVs (*Rltr1B*, *Rltr45, Erv-L, Erv3 Mta*)*, Line-1*, *Rig-I Irf7,* and *Psmb9* in RM1 cells as determined by qRT-PCR following 3 days of treatment with the indicated doses of MeT. Gene expression was normalized to *Hprt.* Vehicle for each gene was set to 1. Error bars are SEM; *P* values (treatment compared to vehicle at each timepoint) were determined using ANOVA and Dunnett multiple comparisons tests (*, *P* < 0.05; **, *P* < 0.01; ***, *P* < 0.001; ****, *P* < 0.0001). **F,** ICS assay demonstrating IFNγ^+^ in CD8^+^ T cells. T cells were cocultured with RM1 cells treated with indicated doses of MeT or DHT for 3 days. Vehicle control for each AR ligand was set to 1. Error bars are SEM; *P* values (treatment compared with vehicle at each timepoint) were determined using ANOVA and Dunnett multiple comparisons tests (*, *P* < 0.05; **, *P* < 0.01; ***, *P* < 0.001; ****, *P* < 0.0001).

IFN-mediated antiviral defence signaling is associated with increased immunogenicity of solid tumors and improved responses to immune checkpoint therapy (59, 61, 67–69). Indeed, we found that MeT treatment caused increased expression of MHC class I antigen processing and presentation genes over a period of 3–6 days (Fig. 7A). Moreover, AR activity was positively correlated with class I (but not class II: *r* = 0.003 and *P* = 0.96 for TCGA; *r* = 0.174 and *P* = 0.06 for SU2C) MHC-mediated antigen processing and presentation in TCGA and SU2C cohorts (Fig. 7B).

To determine whether type I IFN-driven modulation of immune signaling in prostate cancer in response to MeT influences T-cell function, we utilized the murine RM1 model of CRPC (18). We first confirmed that RM1 cells expressed AR (Fig. 7C) and were growth inhibited by MeT and DHT (Fig. 7D; Supplementary Fig. S5A), which collectively highlight the suitability of this model as a tool to understand the impact of potent androgen treatment on prostate cancer biology. Mirroring the findings from human prostate cancer cell lines, MeT increased expression of ERVs (murine *ERV-L*, *MTA*, *RLTR1B,* and *RLTR45*), *LINE-1* elements, *Rig-I* and *Irf7* in RM1 cells (Fig. 7E). Despite DHT having equivalent growth-suppressive effects, it did not influence expression of transposable elements, *Rig-I* or IFN pathway genes (Supplementary Fig. S5B). We next used an *ex vivo* coculture system to assess whether MeT-mediated alterations to the RM1 cellular phenotype could lead to T-cell activation. While DHT treatment had no effect on T-cell response, MeT treatment of RM1 cells increased the immunogenicity of RM1 cells, resulting in enhanced CD8^+^ T-cell recognition and functional cytokine production measured using an ICS assay (Fig. 7F).

## Discussion

Although the mainstay treatment for advanced prostate cancer relies on suppression of AR activity, there is accumulating evidence that potent activation of AR by treating patients with CRPC with high doses of testosterone can also be of therapeutic benefit. The molecular mechanisms underlying this apparent paradox remain to be fully elucidated. Here, by using a synthetic and highly potent androgen, MeT, we provide new insights into the consequences of hyperactivation of AR in prostate cancer.

Molecular dissection of AR activity revealed that MeT elicits remarkably similar activity to DHT in terms of qualitative effects on the transcriptome and AR cistrome. Thus, MeT does not appear to “reprogram” the activity of AR, which has been postulated as a mechanism underlying growth-inhibitory activity of high dose DHT and the synthetic androgen R1881 (14, 15). Instead, our data indicate that MeT more potently regulates canonical (i.e., DHT-regulated) AR signaling: (i) 99% of androgen-regulated genes were altered more strongly with MeT; (ii) almost all AR DNA binding events were stronger and/or more stable in the presence of MeT; and (iii) MeT was more potent in transcriptional activation assays using an androgen-responsive reporter gene construct, which is consistent with previous work (70). The molecular basis for the greater potency of MeT compared with DHT is unknown but we favor the view that it largely relates to increased stability arising from the C17α methyl group (71). However, we cannot rule out the possibility that alterations to AR-bound MeT, such as altered coregulator interactions, may increase its potency.

The potency of MeT was manifested by robust regulation of DNA damage/repair and replication pathways and gene sets that are known to respond to high doses of T and DHT and have been suggested to (at least partly) underpin growth suppression and cell death caused by high-dose androgen treatment (48–50). Interestingly, despite strong downregulation of DNA repair genes, we did not observe increased staining of the DNA damage marker γH2AX in response to MeT. Increased DNA damage has been purported to be a mechanism by which high-dose androgen treatment causes cell death (48), synergizes with agents that inhibit DNA repair (48) and elicits therapeutic responses in PDXs (72) or patients with defective homology-directed repair (HDR; refs. 73, 74). This concept has led to a prevailing belief that HDR gene defects could be useful predictive biomarkers of BAT (48) and that combining BAT with DNA-damaging therapies, such as radiotherapy (e.g., NCT04704505), is a rational therapeutic strategy. In contrast, other studies failed to detect heightened DNA damage in response to high doses of physiologic or synthetic (i.e., R1881) androgens (75), and a recent analysis of BAT clinical trials failed to demonstrate improved progression-free survival in patients with HDR gene defects (76). We propose that definitively establishing the relevance of DNA damage as a mediator of therapeutic response to androgen therapies (including MeT) is imperative to maximize clinical impact.

In addition to its effects on DNA replication and repair pathways, our data revealed that MeT caused a major shift in the expression profile of TEs, including ERVs such as ERV3-1 and HERV-K. This occurred subsequent to downregulation of DNMT enzymes, including DNMT and DNMT3b, and reduction of DNA methylation at LINE-1 elements. Given the well-established role of DNA methylation in suppressing the expression (and mobility) of ERVs and other TEs (57), we propose that inhibition of DNMTs is a key mechanism underlying our observation. A negative association between the expression/activity of AR and DNMTs has been reported previously (e.g., ref. 77), but the molecular underpinnings of this phenomenon are not known. One plausible explanation is that hyperactive AR decreases DNMT expression via its interplay with Rb and E2F. More specifically, it has been reported that high-dose androgen treatment leads to AR and Rb binding to, and transcriptionally repressing, a series of E2F-regulated genes involved in DNA replication (49), a finding that we recapitulated with MeT. Because DNMT1 is a well-established target of E2F (78, 79), it is reasonable to expect that it would be downregulated by AR-mediated perturbation of the Rb/E2F1 axis. Such a mechanism is reminiscent of an earlier study demonstrating that the CDK4/6 inhibitor abemaciclib reduces E2F activity and thereby decreases DNMT1 expression (80).

Upregulation of ERVs can result in accumulation of cellular dsRNA (59), which is sensed by PRRs (i.e., RIG-I, STING) that signal via MAVS/TBK-1 to activate IFN signaling. We propose that this “viral mimicry” response is a key mechanism by which MeT activates IFN, although we cannot rule out the possibility that host long non-coding RNAs and/or miRNAs induced by MeT play a role in RIG-I activation, as has been described recently (81). Viral mimicry is thought to be an important mediator of tumor innate immunity in response to epigenetic therapies such as DNMT inhibitors, histone deacetylase inhibitors, CDK4/6 inhibitors and EZH2 inhibitors (59, 60, 69, 80, 82, 83). In support of this, we demonstrated that MeT enhanced the immunogenicity of murine prostate cancer cells leading to elevated T-cell responses in a coculture system, providing *in vitro* evidence that a viral mimicry response induced by this androgen could modulate the tumor immune microenvironment.

Prostate cancer is recognized as an immunologically “cold” cancer type based on its tumor microenvironment (i.e., few infiltrating cytotoxic T cells and a predominance of immunosuppressive cells, such as regulatory T cells and M2 macrophages), low immunogenicity and downregulation of MHC class I antigen-processing/presenting machinery in tumor cells (84). These characteristics likely explain the limited impact of immunotherapies in this disease to date (84). A cellular immunotherapy, Sipuleucel-T, is approved for men with metastatic CRPC but only confers a survival benefit of approximately 4 months (85). Similarly, multiple trials of immune checkpoint inhibitors (ICI) have failed to demonstrate overall survival benefits (84), although some patients have experienced extraordinary responses to this treatment strategy (74, 86). With this background in mind, there is considerable interest in developing combinatorial treatment strategies that would sensitize CRPC tumors to immunotherapy. Our study found that MeT enhanced expression of MHC class I genes and increased T cell activity, suggesting that this regulator of viral mimicry could increase tumour cell immunogenicity, which is critical to improve response to ICIs. In support of this concept, a recent study found that inhibition of EZH2 activated a dsRNA–STING–IFN stress response that increased intratumoral trafficking of activated CD8^+^ T cells and sensitized prostate cancer cells to PD-1 checkpoint blockade (69). Moreover, there is evidence that both AR activation (i.e., BAT) and AR inhibition (i.e., enzalutamide) could sensitize tumors to PD-1 inhibitors, albeit in very small studies (74, 86). Whether response to ICIs in patients previously treated with BAT is a result of viral mimicry is an enticing possibility that warrants further investigation, either using preclinical models and/or by molecular analysis of samples from patients being treated by BAT/ICI in ongoing clinical trials (e.g., COMBAT-CRPC, NCT03554317).

Immunologic priming by BAT has been hypothesized to be a consequence of androgen-mediated DNA damage, which can be sensed by the dsDNA sensor protein cGAS that can in turn activate IFN signaling (74). At least two lines from our study of evidence suggest that this hypothesis should be modified to consider dsRNA as an alternative trigger of IFN signaling. First, MeT (and to a lesser extent DHT) induced ERVs, RIG-I, and MAVS and caused accumulation of dsRNA. Second, we did not observe a substantial increase in DNA damage—using γH2AX as a molecular marker of DNA damage—in response to MeT treatment in LNCaP cells. However, the absence of γH2AX foci does not preclude MeT-mediated DNA damage, nor did we specifically measure cytoplasmic DNA. In addition, STING, which is traditionally thought of as a sensor of cytoplasmic DNA, was induced by MeT, although it must be noted that emerging evidence suggests that this factor also plays a key role in dsRNA-based immune responses (62, 69). In short, it is plausible that the multifactorial impact on transcription and genome organization caused by MeT (or high doses of DHT/T) would result in both dsRNA accumulation and DNA damage, both of which could elicit viral mimicry and IFN signaling.

An important question that still needs to be addressed is whether, and to what extent, activation of IFN signaling contributes to MeT-mediated suppression of prostate cancer cell growth. Type I IFNs can elicit cell-cycle arrest and apoptosis in malignant cells (87), therefore it is possible that induction of this pathway at least partly explains the efficacy of MeT. However, MeT (and high doses of T/DHT) cause growth suppression within 1–2 days, whereas we observed induction of *IFNβ* and *IRF7* 3–6 days after treatment, an observation that is consistent with a stepwise activation of IFN involving epigenomic remodeling, ERV transcription and sensing of dsRNA. Moreover, growth suppression of RM1 cells by MeT and DHT was equivalent, even though the latter hormone did not induce ERVs or the IFN pathway. These observations argue against viral mimicry and IFN pathway activation playing a major role in the growth-inhibitory effects of MeT, at least when prostate cancer cells are grown *in vitro*. Future *in vivo* studies carried out in the context of antagonism or ablation of viral mimicry effectors (e.g., RIG-I, IFNβ) could resolve this outstanding question.

A consistent finding throughout our study was that MeT exhibited greater potency—in terms of prostate cancer cell growth inhibition, AR DNA binding and transcriptional activity and viral mimicry responses—than DHT. MeT has been reported to have reduced affinity, when compared with DHT, for both the rat AR ligand-binding domain (88, 89) and cytosolic fractions from rat prostate (90). However, the main pathway for metabolism of testosterone and its derivatives in prostate cancer cells is via glucuronidation (91), and MeT is very poorly glucuronidated by human glucuronyl-transferases (92). With these early biochemical studies in mind, we propose that the stronger androgenic effects elicited by MeT relate to its increased stability compared with DHT. Moreover, we hypothesize that the increased potency of MeT, as opposed to a differential mode of action, explains why activation of IFN has not been observed in previous studies aimed at dissecting the mode of action of high-dose DHT and other androgens (i.e., R1881; refs. 48, 50). This hypothesis is supported by the observation that DHT elicited effects on ERVs, dsRNA production and IFN signaling that were qualitatively analogous to those mediated by MeT but were in almost all cases weaker. In short, we postulate that a certain threshold of AR activation, in terms of both strength and duration, is required to activate a viral mimicry response and that such a threshold can be more readily reached with stable synthetic androgens such as MeT.

Whether the anabolic-androgenic steroid MeT could be harnessed as a therapeutic for advanced prostate cancer, either in combination with ADT in a “BAT-like” therapy or as an immunotherapy-sensitizing agent, is an intriguing question. Current medical recommendations suggest that MeT should be explicitly avoided in men with prostate cancer but these are based on the historical viewpoint that androgens promote tumor progression, which is overly simplistic in the era of SupraT/BAT as a rational and safe treatment for CRPC. Although MeT has a range of medical uses, including to treat delayed puberty in males (93), as a component of menopausal hormone therapy in women (94) and, historically, as a treatment for breast cancer (95), it is no longer commonly used in these clinical scenarios. Drawbacks of MeT include high estrogenicity, due to its efficient aromatization into the potent and stable estrogen 17α-methylestradiol (96), and hepatotoxicity (97). The enhanced potency and stability of MeT could provide advantages over testosterone in BAT. Of course, other AR ligands may be even more effective than MeT in terms of prostate cancer growth suppression and/or modulation of immune responses. In this respect, selective AR modulators (SARM) are of interest (98) because it is conceivable that some may possess the requisite androgenic anti-growth and immunomodulatory activities in prostate cancer cells and favorable anabolic properties in other tissues. Indeed, a recent study found that SARMs with potent growth-suppressive activity in prostate cancer activated the transcriptional activity of AR in a manner highly concordant to that of steroidal androgens, although effects on innate immune signaling were not reported in this study (99). Thus, we propose that research beyond the physiologic androgens testosterone and DHT will be required to fully capitalize on the therapeutic potential of AR activation.

Our investigations have revealed a novel consequence of potent activation of AR by MeT in prostate cancer cells. We propose that this work will expose new avenues of research aimed at elucidating interplay between androgenic and immune responses in the prostate and potentially facilitate the development of new hormonal strategies to sensitize prostate cancer to immunotherapies.

## Supplementary Material

Supplementary Figures S1-5 and Table S1 Supplementary Figures 1-5 and Table S1

Supplementary Dataset S1 Supplementary ChIP-seq data analysis

Supplementary Dataset S2 Supplementary RNA-seq data analysis

Key Resources Table KRT1 Key Resources Table
