# Supplementary material for: Potent Stimulation of the Androgen Receptor Instigates a Viral Mimicry Response in Prostate Cancer
Source: Cancer Res Commun. 2022 Jul 25;2(7):706–24. doi: 10.1158/2767-9764.CRC-21-0139 (PMC10010308; doi:10.1158/2767-9764.CRC-21-0139)
Supplement: Supplementary Figures S1-5 and Table S1 — Supplementary Figures 1-5 and Table S1 [file crc-21-0139-s01.pdf]

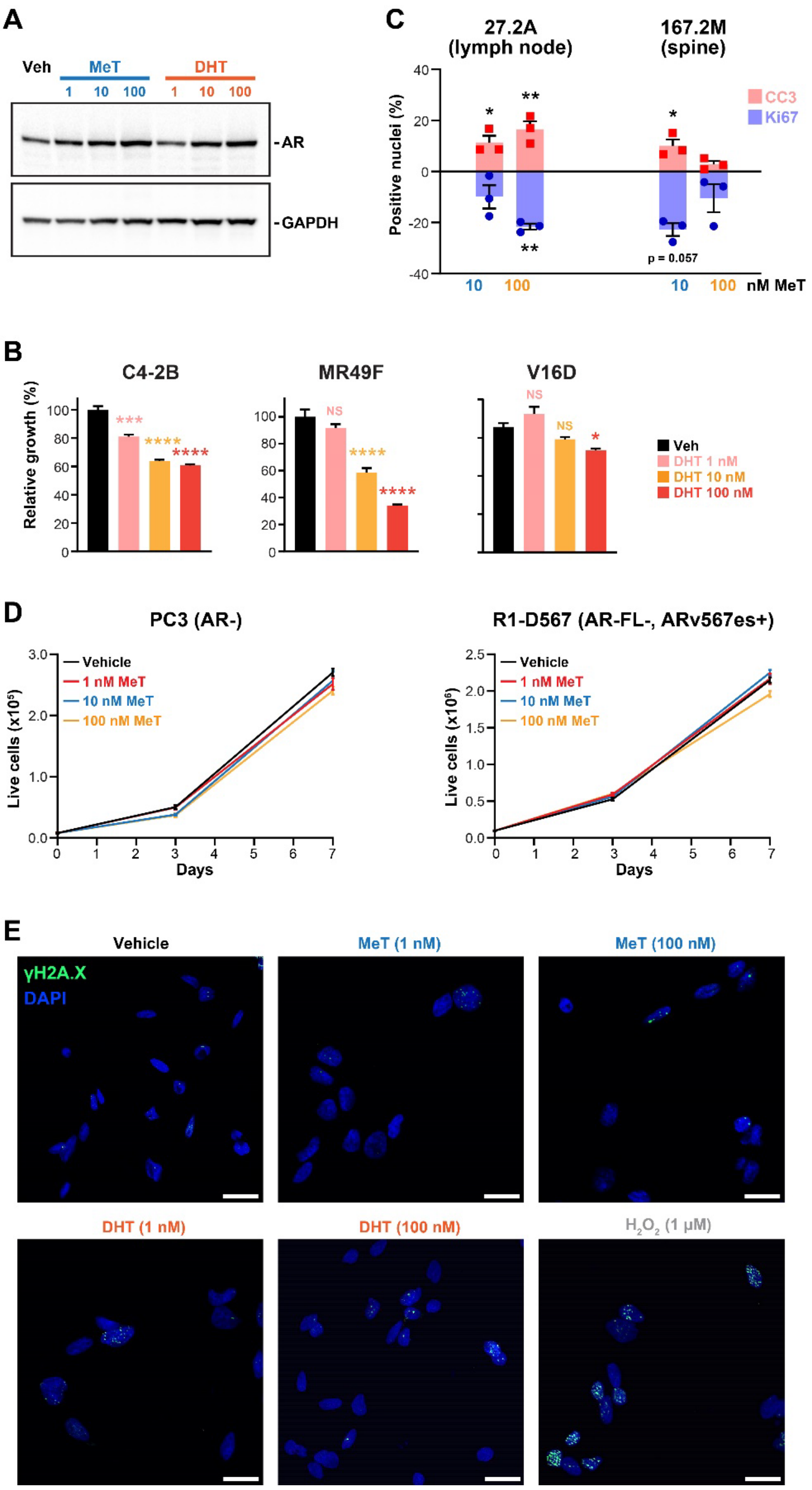

Supplementary Fig. 1. (A) Representative Western blot showing AR protein levels following treatment of LNCaP cells with the indicated doses of MeT or DHT or vehicle control for 24. GAPDH is shown as a loading control. **(B)** Effect of DHT on the growth of CRPC models of PCa (C4-2B, MR49F and V16D), as determined by Trypan blue growth assay. P values (using

day 7 data) were determined using ANOVA and Dunnett's multiple comparisons tests (*, $p < 0.05$; ***, $p < 0.001$; ****, $p < 0.0001$). NS, not significant. **(C)** MeT has anti-tumor activity against patient-derived xenografts (PDXs; 27.2A and 167.2M) grown *ex vivo*. PDX-derived tumor fragments were treated for 48 h with the indicated doses of MeT. Graphs represent average changes in the percentage of cells positive for cleaved caspase 3 (CC3) or Ki67 versus the vehicle control. Error bars are ± SEM. P values were determined using t tests of absolute Ki67 and Casp3 values in each treated group compared to its matching vehicle control from the same PDX tissue (*, $p < 0.05$; **, $p < 0.01$). **(D)** Methyl-testosterone does not affect the growth of PC3 or R1-D567 prostate cancer cells. Cell growth was measured using Trypan blue growth assays. Error bars are ± SEM. AR-FL, AR full-length; ARv567es, AR variant 567es. **(E)** Representative images of γH2AX immunofluorescence (related to Fig. 2G). Scale bar = 20 μM.

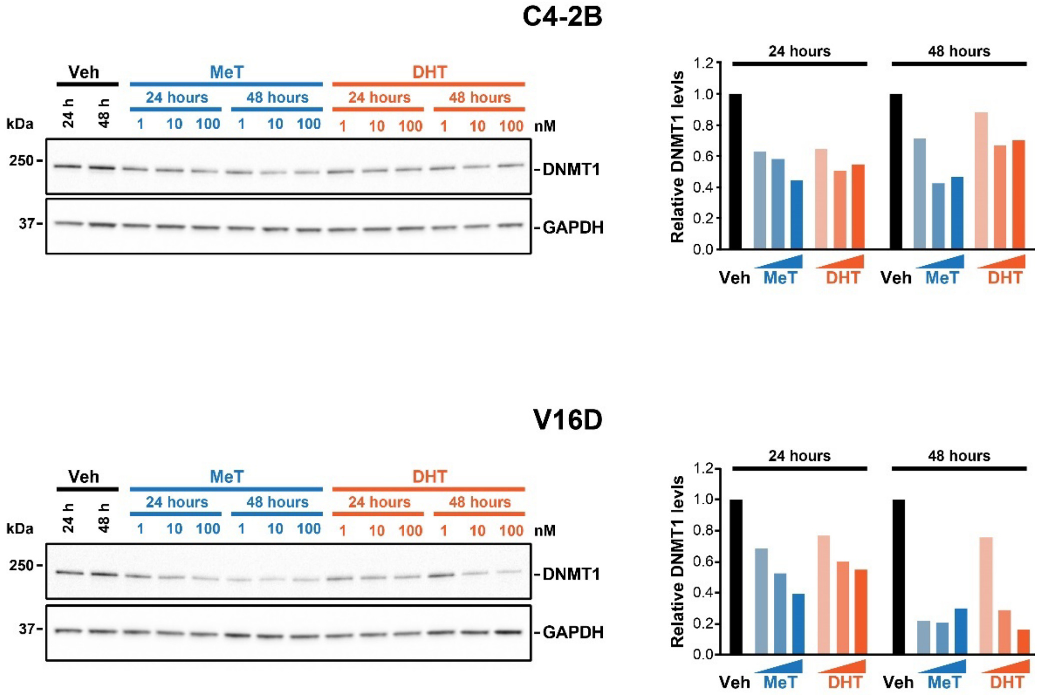

Supplementary Fig. 2. Methyl-testosterone down-regulates DNMT1. Representative Western blots showing DNMT1 protein levels following treatment of C4-2B (top) and V16D (bottom) cells with the indicated doses of MeT or DHT or vehicle control for 24 and 48 hours. GAPDH is shown as a loading control. Quantification of DNMT1 protein (normalised to GAPDH) is shown on right.

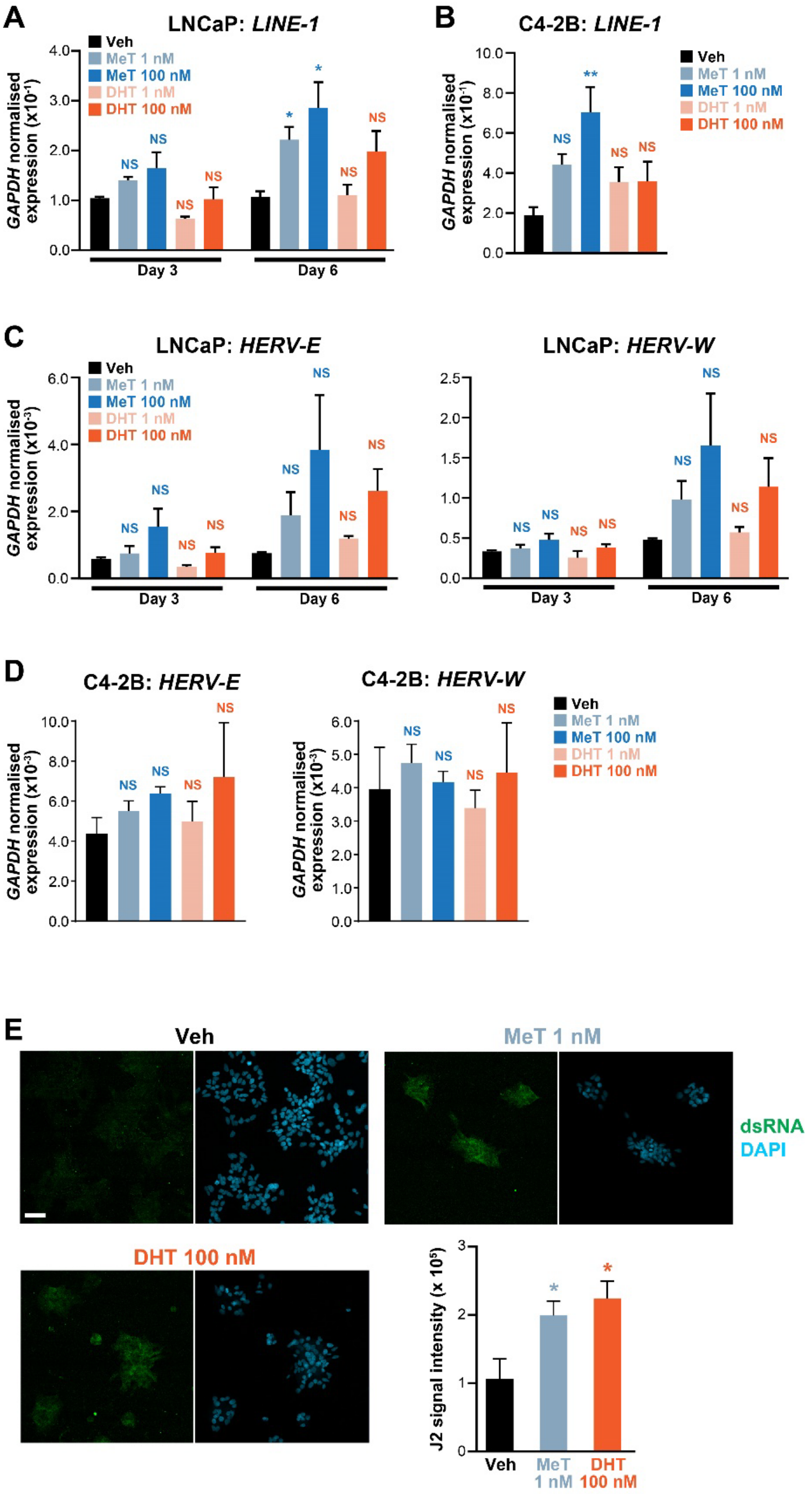

Supplementary Fig. 3. Regulation of transposable element expression by methyl-testosterone. (A-B) Expression of *LINE-1*, as determined by qRT-PCR, following treatment with MeT or DHT in LNCaP (A) and C4-2B (B; 3 day treatment) cells. Expression of *LINE-1* was normalized to *GAPDH*. Error bars are SEM; P values (treatment compared to vehicle) were determined using ANOVA and Dunnett's multiple comparisons tests (*, $p < 0.05$; **, $p < 0.01$). **(C)** Expression of HERV-E and HERV-W, as determined by qRT-PCR, following treatment with MeT or DHT in LNCaP cells. Expression of ERVs was normalized to *GAPDH*. Error bars are SEM; significance (treatment compared to vehicle) was determined using

ANOVA and Dunnett's multiple comparisons tests. **(D)** Expression of HERV-E and HERV-W, as determined by qRT-PCR, following treatment with MeT or DHT for 3 days in C4-2B cells. Expression of ERVs was normalized to *GAPDH*. Error bars are SEM; significance (treatment compared to vehicle) was determined using ANOVA and Dunnett's multiple comparisons tests. **(E)** Quantitation of cellular dsRNA in VCaP cells by immunofluorescent staining with J2 monoclonal antibody following 3 days of treatment with MeT or DHT. Error bars are SEM; P values (treatment compared to vehicle) were determined using ANOVA and Dunnett's multiple comparisons tests (****, $p < 0.0001$). Representative images of J2 immunofluorescence are shown (J2 signal, representing cellular dsRNA, is in green; nuclei were counterstained with DAPI, in blue). Scale bar = 50 μM.

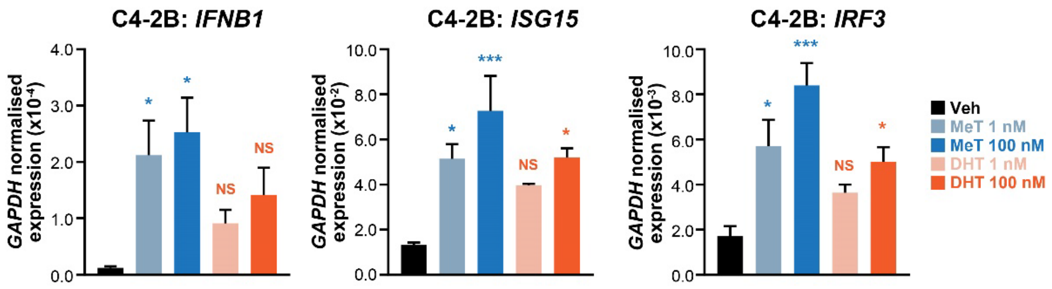

Supplementary Fig. 4. Induction of IFN signalling by methyl-testosterone in C4-2B cells. Expression of IFNβ (encoded by *IFNB1*), *ISG15* and *IRF3*, as determined by qRT-PCR, following treatment with MeT or DHT for 6 days in C4-2B cells. Expression of genes was normalized to *GAPDH*. Error bars are SEM; P values (treatment compared to vehicle) were determined using ANOVA and Dunnett's multiple comparisons tests (*, $p < 0.05$; ***, $p < 0.001$).

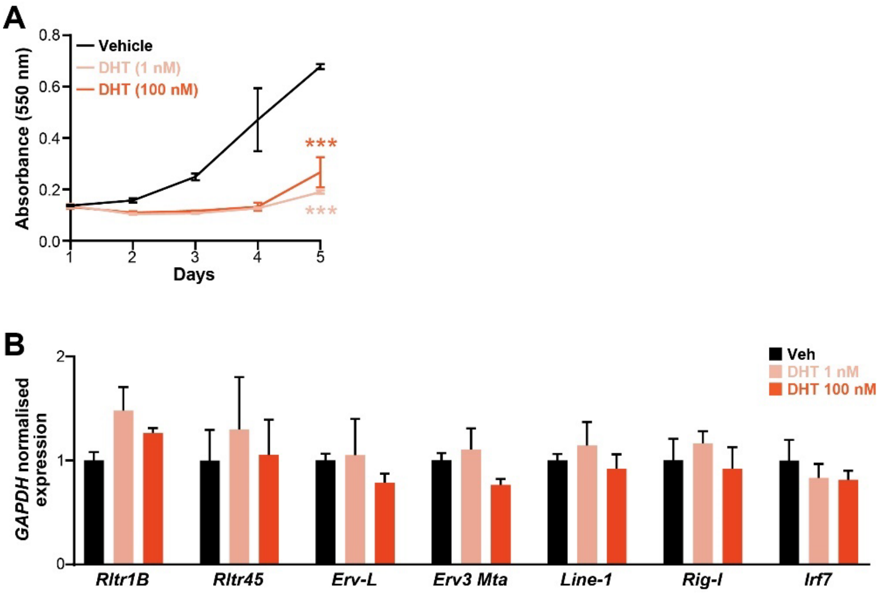

Supplementary Fig. 5. Effects of DHT on RM1 murine model of prostate cancer. (A) DHT suppresses the growth of RM1 cells, as determined by Sulforhodamine B colorimetric assay Mean absorbance (550 nm) is shown at the indicated time-points; error bars are ± SEM. P values were determined using unpaired t tests at day 5 (***, $p < 0.001$). **(B)** Expression of ERVs (*Rltr1B*, *Rltr45, Erv-L, Erv3 Mta*), *LINE-1*, *RIG-I IRF7* and *ISG15* in RM1 cells as determined by qRT-PCR following 3 days of treatment with the indicated doses of DHT. Gene expression was normalized to *Hprt.* Vehicle for each gene was set to 1. Error bars are SEM; P values (treatment compared to vehicle at each time-point) were determined using ANOVA and Dunnett's multiple comparisons tests (no significant differences for any transcripts with either dose of DHT).

Table S1. Primers used in this study

Primer	Sequence	Species
STING-Fwd	AGCATTACAACAACCTGCTACG	Human
STING-Rev	GTTGGGGTCAGCCATACTCAG	Human
ERV3-env-Fwd	CCATGGGAAGCAAGGGAACT	Human
ERV3-env-Rev	CTTTCCCCAGCGAGCAATAC	Human
HERV-W-Fwd	TGAGTCAATTCTCATACCTG	Human
HERV-W-Rev	AGTTAAGAGTTCTTGGGTGG	Human
HERVE Fwd	GGTGTCACTACTCAATACAC	Human
HERVE-Rev	GCAGCCTAGGTCTCTGG	Human
HERVF-Fwd	CCTCCAGTCACAACAACTC	Human
HERVF-Rev	TATTGAAGAAGGCGGCTGG	Human
ERVL-Fwd	ATATCCTGCCTGGATGGGGT	Human
ERVL-Rev	GAGCTTCTTAGTCCTCCTGTGT	Human
HERV-K-Fwd	ATTGGCAACACCGTATTCTGCT	Human
HERV-K-Rev	CAGTCAAAATATGGACGGATGGT	Human
DNMT1-Fwd	GCGTTCCGGCTGAACAAC	Human
DNMT1-Rev	GCATCTCCACGTCTCCCT	Human
EZH2-Fwd	GTGGAGAGATTATTTCTCAAGATG	Human
EZH2-Rev	CCGACATACTTCAGGGCATCAGCC	Human
B2M-Fwd	TGACTTTGTCACAGCCCAAG	Human
B2M-Rev	AGCAAGCAAGCAGAATTTGG	Human
HLA-A-Fwd	GGCCCTGACCCAGACCTG	Human
HLA-A-Rev	GCACGAACTGCGTGTCGTC	Human
HLA-B-Fwd	ACTGAGCTTGTGGAGACCAGA	Human
HLA-B-Rev	GCAGCCCCTCATGCTGT	Human
HLA-C-Fwd	CTGGCCCTGACCGAGACCTG	Human
HLA-C-Rev	CGCTTGTACTTCTGTGTCTCC	Human
IFN-β-Fwd	GCCATCAGTCACTTAAACAGC	Human
IFN-β-Rev	GAAACTGAAGATCTCCTAGCCT	Human
ISG15-Fwd	CCTTCAGCTCTGACACC	Human
ISG15-Rev	CGAACTCATCTTTGCCAGTACA	Human
IRF7-Fwd	GTGGACTGAGGGCTTGTAG	Human
IRF7-Rev	TCAACACCTGTGACTTCATGT	Human
MAVS-Fwd	AGGAGACAGATGGAGACACA	Human
MAVS-Rev	CAGAACTGGGCAGTACCC	Human
RIG-I-Fwd	CCAGCATTACTAGTCAGAAGGAA	Human
RIG-I-Rev	CACAGTGCAATCTTGTCATCC	Human
Irf7-Fwd	CCACACCCCCATCTTCGA	Mouse
Irf7-Rev	CCTCCGAGCCCGAAACTC	Mouse
Psmb9-Fwd	TAGTAGCTGGCTGGGACCAA	Mouse
Psmb9-Rev	GATGGTAAAGGGCTGTCGAA	Mouse
Hprt-Fwd	GGCCAGACTTTGTTGGATTT	Mouse

Hprt-Rev	ACTGGCAACATCAACAGGACT	Mouse
Ddx58 (Rig-I)-Fwd	AAGAGCCAGAGTGTCAGAATCT	Mouse
Ddx58 (Rig-I)-Rev	AGCTCCAGTTGGTAATTTCTTGG	Mouse
Line1-Fwd	GGACCAGAAAAGAAATTCCTCCCG	Mouse
Line1-Rev	CTCTTCTGGCTTTCATAGTCTCTGG	Mouse
ERV-Mta -Fwd	TCTGTGGGATGTTGTGTAGGAG	Mouse
ERV-Mta-Rev	CCACAGATCTTCACAATCCAAA	Mouse
Rltr1B-Fwd	GGTCCACACAAACACCTACCTT	Mouse
Rltr1B -Rev	TTTGAGATACACCCTTCGAGGT	Mouse
Rltr45-Fwd	ACCTTGGACCTTTCTCAATACAT	Mouse
Rltr45-Rev	GACCTCCTCCTAATAACCAAATG	Mouse
