# Supplementary material for: Potent Stimulation of the Androgen Receptor Instigates a Viral Mimicry Response in Prostate Cancer
Source: Cancer Res Commun. 2022 Jul 25;2(7):706–24. doi: 10.1158/2767-9764.CRC-21-0139 (PMC10010308; doi:10.1158/2767-9764.CRC-21-0139)
Supplement: Key Resources Table KRT1 — Key Resources Table [file crc-21-0139-s04.docx]

| **Key Resources Table** | | | | |
| --- | --- | --- | --- | --- |
| **Reagent type (species) or resource** | **Reagents** | **Source or reference** | **Identifiers** | **Additional information** |
| chemical compound, drug | iScript cDNA synthesis kit | BioRad | Cat#: 170-8891 |  |
| chemical compound, drug | Methanol | Chem Supply | Cat#: MA004-2.5L-P |  |
| chemical compound, drug | TRIZOL Reagent | Sigma Aldrich | Cat#: T9424 |  |
| chemical compound, drug | Poly(I:C) (LMW) / LyoVec™ | Invivogen | Cat#: tlrl-picwlv |  |
| chemical compound, drug | \| 17α-Methyltestosterone/ Mesterone \| \| --- \| | \| Sigma Aldrich \| \| --- \| | Cat#: M7252 |  |
| chemical compound, drug | Dihydrotestosterone | Sigma Aldrich | Cat#: 521-18-6 |  |
| cell line (*Homo-sapiens*) | LNCaP | ATCC | ATCC CRL-1740  (RRID:CVCL_1379) |  |
| cell line (*Homo-sapiens*) | VCaP | ATCC | ATCC CRL-2876  (RRID:CVCL_2235) |  |
| cell line (*Homo-sapiens*) | PC3 | ATCC | ATCC CRL-7934  (RRID:CVCL_0035) |  |
| cell line (*Homo-sapiens*) | 22RV1 | ATCC | ATCC CRL-2505  (RRID:CVCL_1045) |  |
| cell line (*Homo-sapiens*) | V16D | PMID: 27046225 | Kind gift from Prof. Amina Zoubeidi |  |
| cell line (*Homo-sapiens*) | MR49F | PMID: 27046225 | Kind gift from Prof. Amina Zoubeidi |  |
| cell line (*Homo-sapiens*) | C4-2B | ATCC | Cat# CRL-3315, RRID:CVCL_4784 |  |
| antibody | mouse anti-dsRNA | SCICONS | Cat# 10010200, RRID:AB_2651015 | Used at 1:500 for immunofluorescence |
| antibody | MHC class I | BioLegend | Cat# 114602, RRID:AB_313593 | Used at 1:1000 for flow cytometry |
| antibody | p-STAT1 | Cell Signaling Technology | Cat# 9170, RRID:AB_330373 | Used at 1:1000 for Western blotting |
| antibody | STAT1 | Cell Signaling Technology | Cat# 9175, RRID:AB_2197984 | Used at 1:1000 for Western blotting |
| antibody | Phospho-Rb (Ser780) Antibody | Cell Signaling Technology | Cat# 9307, RRID:AB_330015 | Used at 1:1000 for Western blotting |
| antibody | Rb (4H1) Mouse mAb | Cell Signaling Technology | Cat# 9309, RRID:AB_823629 | Used at 1:1000 for Western blotting |
| antibody | TBK1/NAK Antibody | Cell Signaling Technology | Cat# 3013, RRID:AB_2199749 | Used at 1:1000 for Western blotting |
| antibody | Phospho-TBK1/NAK (Ser172) (D52C2) XP® | Cell Signaling Technology | Cat# 13498, RRID:AB_2798237 | Used at 1:1000 for Western blotting |
| antibody | Tri-Methyl-Histone H3 (Lys27) (C36B11) | Cell Signaling Technology | Cat# 4395, RRID:AB_11220433 | Used at 1:1000 for Western blotting |
| antibody | Anti-phospho-Histone H2A.X | Millipore | Cat# 05-636, RRID:AB_309864 | Used at 1:1000 for immunofluorescence |
| antibody | GAPDH | Millipore | Cat# AB9132, RRID:AB_347661 | Used at 1:5000 for Western blotting |
| antibody | RIG-I | Santa Cruz Biotechnology | Cat# sc-376845,  RRID:AB_2732794 | Used at 1:1000 for Western blotting |
| antibody | AR | Abcam | Cat# ab108341,  RRID:AB_10865716 | Used at 1:1000 for Western blotting |
